# Healthcare Professionals’ Perspectives on Interdisciplinary Teamwork for Falls Prevention Among Older Adults in Hospital Settings: A Scoping Review

**DOI:** 10.3390/healthcare14213658

**Published:** 2026-10-28

**Authors:** Aisha Alenzy, James McMahon, Gary Mitchell, Gillian Prue

**Affiliations:** 1 School of Nursing and Midwifery, Queen’s University Belfast, Belfast BT7 1NN, UK; j.mcmahon@qub.ac.uk (J.M.); gary.mitchell@qub.ac.uk (G.M.); g.prue@qub.ac.uk (G.P.); 2 College of Nursing, Najran University, Najran 11001, Saudi Arabia

**Keywords:** older adults, scoping review, interdisciplinary teamwork, falls prevention, hospital, interprofessional collaboration, patient safety

## Abstract

**Background/Objectives.** Falls among hospitalized older adults remain a persistent patient-safety concern. Interdisciplinary teamwork is widely recommended for falls prevention, but how it is conceptualized and operationalized in hospital settings has not been systematically mapped. This scoping review aimed to map healthcare professionals’ perspectives on this teamwork. **Methods.** The review followed the Arksey and O’Malley framework and the Joanna Briggs Institute methodology and was reported in accordance with PRISMA-ScR. The protocol was registered retrospectively on the Open Science Framework. Five databases were searched on 10 August 2026 for peer-reviewed studies published in English or Arabic between 2013 and 2025. Reference lists of included studies were checked, and a supplementary MEDLINE sensitivity search was conducted. Two reviewers independently screened database records and assessed all full texts, and data were charted and analyzed descriptively. **Results.** Twenty-one studies from eight countries were included. The evidence was organized into four descriptive categories, namely roles and shared accountability, communication and coordination, organizational influences, and interprofessional education and patient engagement. Healthcare professionals valued interdisciplinary teamwork, but shared accountability was not consistently operationalized, communication barriers were often related to information systems and workflow, and organizational conditions frequently constrained collaborative practice. **Conclusions.** The evidence base is small and predominantly descriptive, and no study reported a statistically significant reduction in falls. Future research should examine how teamwork processes relate to falls outcomes across diverse hospital systems.

## 1. Introduction

Falls among hospitalized older adults remain a persistent patient-safety concern. An estimated 684,000 fatal falls occur globally each year, and adults aged 60 years and over experience the highest rates of fall-related mortality and morbidity [1]. This scoping review examines falls occurring during hospital admission. In hospital settings, falls are associated with fractures, head injuries, loss of independence, prolonged length of stay, and increased healthcare costs [2]. Hospital falls are complex and multifactorial, reflecting interactions among physiological, environmental, and care-process factors. Their prevention therefore requires coordinated input from multiple professional groups rather than the efforts of a single discipline [3]. Despite advances in clinical practice and research, falls remain an ongoing challenge in hospitals, and evidence on effective prevention strategies continues to evolve [4,5].

Multifactorial interventions incorporating patient education, environmental modification, and staff training have been shown to reduce falls in hospital settings, although the evidence remains mixed and implementation varies considerably across contexts [3,5]. A Cochrane systematic review found that multifactorial interventions may reduce the rate of falls in hospitals but rated the certainty of this evidence as low [3]. A recent systematic review and meta-analysis of hospital falls interventions found that effectiveness varied with local implementation and sustainability [5]. The World Falls Guidelines, developed through international consensus, recommend multifactorial assessment and tailored interventions involving interprofessional and patient-centered approaches as core components of falls prevention for older adults [6]. The Safe Recovery Programme, which was associated with a 40% lower rate of falls on hospital rehabilitation units when individualized patient education was combined with staff training and feedback [7], and the 6-PACK program, which did not reduce falls or fall injuries in a cluster randomized trial on acute wards [4], illustrate that hospital-based multifactorial approaches depend on collaborative input from multiple professional groups and that their effects vary across settings.

Despite this reliance on multiple professional groups, the role of interprofessional collaboration in hospital falls prevention has received limited empirical attention. The World Health Organization Framework for Action on Interprofessional Education and Collaborative Practice defines collaborative practice as occurring when multiple health workers from different professional backgrounds collaborate with patients, families, and communities to deliver comprehensive care [8]. In this review, interdisciplinary teamwork refers to collaborative practice involving two or more healthcare professionals working across disciplinary boundaries to coordinate care, share information, and make joint decisions related to falls prevention. Because the terms interdisciplinary, interprofessional, and multidisciplinary overlap in the literature, they are used in this review as follows. Interdisciplinary teamwork, as defined above, is the focus of this review. Interprofessional is used for established concepts that describe relationships between members of different professions, such as interprofessional education, interprofessional collaboration, and interprofessional roles, and where the included studies used this term. Multidisciplinary is used where several professions were present or contributed alongside one another without evidence of shared decision-making. A Cochrane review of interprofessional collaboration interventions found that such approaches can improve healthcare processes and outcomes but concluded that the evidence base remains limited and heterogeneous, with the mechanisms through which collaboration produces improvement being poorly understood [9]. In falls prevention specifically, the involvement of multiple professional groups is widely recommended, yet the evidence on how interdisciplinary teamwork is conceptualized, implemented, and experienced by healthcare professionals in hospital settings has not been systematically examined.

A prior scoping review examined education interventions for health professionals on falls prevention in healthcare settings and found that reporting was inconsistent and few studies employed comprehensive education design principles [10]. However, that review focused on the design and quality of education interventions rather than on how interdisciplinary teamwork operates as a process within hospital falls prevention. To the authors’ knowledge, no scoping review has specifically mapped healthcare professionals’ perspectives on how interdisciplinary teamwork is described and operationalized in this context. This review therefore does not evaluate whether falls-prevention interventions are effective. Instead, it maps how interdisciplinary teamwork is perceived, experienced, and operationalized within hospital falls-prevention approaches, a question that effectiveness-focused reviews are not designed to answer. A scoping review is the appropriate method for this question because the literature is heterogeneous in design and conceptual focus, the aim is to map the extent and nature of existing evidence rather than to synthesize effectiveness, and the review seeks to identify gaps in the evidence base rather than to answer a specific clinical question [11,12].

This scoping review aimed to map the existing literature on healthcare professionals’ perspectives on interdisciplinary teamwork for falls prevention among older adults in hospital settings. Its objectives were to (1) explore how healthcare professionals from multiple disciplines perceive and experience interdisciplinary teamwork in preventing falls among older adults in hospital settings, and (2) identify factors that healthcare professionals report as facilitating or constraining interdisciplinary teamwork for falls prevention in hospital environments.

A preliminary search of MEDLINE and the Cochrane Database of Systematic Reviews was conducted in November 2023 before the main database search. It did not identify an existing scoping review that mapped healthcare professionals’ perspectives on interdisciplinary teamwork specifically for falls prevention in hospital settings. Additional checks of PROSPERO, OSF Registries, and JBI Evidence Synthesis in October 2026 identified no registered or published review addressing this question. These findings supported the relevance of the present review.

## 2. Materials and Methods

### 2.1. Protocol and Registration

A review protocol was developed in 2023, before the review commenced, and specified the review question, eligibility criteria, search approach, screening process, and data-charting plan. The review followed the methodological framework proposed by Arksey and O’Malley [11], as enhanced by Levac et al. [13] and refined within the Joanna Briggs Institute (JBI) methodology for scoping reviews [12,14]. Reporting followed the Preferred Reporting Items for Systematic Reviews and Meta-Analyses extension for Scoping Reviews (PRISMA-ScR) guidelines [15]. The completed PRISMA-ScR reporting checklist is provided in the Supplementary Materials (Table S4).

The review then proceeded in the following sequence. A preliminary search of MEDLINE and the Cochrane Database of Systematic Reviews was conducted in November 2023. The protocol initially specified an eligibility period from 2013 to 2023, ending in the year in which the protocol was developed. In March 2026, before the main database search, this period was extended to 2025, the most recent complete publication year at that time, to capture recently published evidence. A complete calendar year was used as the upper limit so that the eligibility period was fixed before the search and did not depend on how many 2026 publications had been indexed by the search date. The main database search was conducted once, on 10 August 2026, and all 664 retrieved records were screened against the final eligibility criteria in a single screening process. Title and abstract screening, full-text screening, and data extraction for the database records were completed in August 2026. The protocol was registered retrospectively with OSF Registries on 16 August 2026, after the database search had been completed (https://doi.org/10.17605/OSF.IO/VCKT8). After data extraction, the age criterion was also refined from adults aged 65 years and over to older adults, for the reason described in Section 2.2. This refinement did not change the inclusion or exclusion of any study, and the registered protocol reflects the final eligibility criteria. The optional consultation stage of the Arksey and O’Malley framework was not undertaken.

### 2.2. Eligibility Criteria

Eligibility criteria were developed using the Population, Concept, and Context (PCC) framework recommended by the Joanna Briggs Institute [12,14]. The review addressed the following question: What is the extent and nature of the existing evidence on healthcare professionals’ perspectives on interdisciplinary teamwork for falls prevention among older adults in hospital settings?

Population. This review focused on healthcare professionals working in hospital environments, including nurses, physicians, pharmacists, and allied health professionals such as physiotherapists, occupational therapists, and social workers. Studies were required to involve participants from two or more professional disciplines or to explicitly examine interdisciplinary dynamics in falls prevention. Studies that also included patients’ or family members’ perspectives were eligible when they met these criteria. The patient population of interest was older adults. The protocol initially specified adults aged 65 years and over, but because the included studies examined healthcare professionals’ perspectives and most did not recruit patients, a numerical age threshold could not be applied at the study level. Studies were therefore eligible when falls prevention was described in relation to older inpatients or hospital settings in which older adults are the main population at risk of falls, such as general medical, surgical, orthopedic, neurology, rehabilitation, and older adults’ mental health wards, and studies were not required to report patient age.

Concept. Interdisciplinary teamwork was defined as collaborative practice involving two or more healthcare professionals working across disciplinary boundaries to coordinate care, share information, and make joint decisions related to falls prevention. Perspectives included views and experiences reported in qualitative studies and perceptions measured through surveys, such as teamwork and patient safety culture questionnaires. Related constructs, including communication, safety culture, and organizational factors, were included when explicitly linked to interdisciplinary teamwork. Studies examining single-discipline knowledge, attitudes, or practices without an interdisciplinary teamwork component were excluded. In practice, studies were eligible at screening when they involved healthcare professionals from more than one discipline in hospital falls prevention or explicitly examined interdisciplinary dynamics. For example, a study limited to physiotherapists’ perspectives on hospital falls prevention was excluded, whereas a study in which interprofessional coordinating teams conducted post-fall huddles was included. The distinction between multidisciplinary participation, in which several professions were present, and interdisciplinary collaboration, involving shared information, coordinated roles, or joint decisions, was not used as a screening filter but was examined during data charting and analysis.

Context. The review was limited to hospital-based care settings, including acute care wards, geriatric units, rehabilitation wards, and other inpatient environments. Studies conducted exclusively in community, residential care, primary care, or home-based settings were excluded. No geographical restrictions were applied. Only peer-reviewed empirical studies published in English or Arabic between 2013 and 2025 were included. Arabic was included because the lead author is fluent in Arabic and the wider program of research of which this review forms part concerns hospitals in Saudi Arabia, so Arabic-language studies from the region were of particular relevance. These restrictions were applied to focus the review on contemporary, peer-reviewed empirical evidence and to enable feasible screening and data charting in the review languages. Grey literature was not searched and may therefore contain additional implementation evidence.

### 2.3. Search Strategy

A three-step search strategy was employed, in line with JBI guidance, followed by a supplementary sensitivity search. First, a limited search of MEDLINE and CINAHL was conducted to identify relevant index terms and text words in titles and abstracts. Second, the comprehensive search was conducted on 10 August 2026 across MEDLINE via EBSCOhost, CINAHL via EBSCOhost, PsycINFO via Ovid, the Cochrane Library, and Europe PMC. Date limits were applied in MEDLINE and CINAHL, covering 1 July 2013 to 31 July 2025, and in PsycINFO, covering 2013 to 2025. The Cochrane Library and Europe PMC searches were not date-limited, so the publication date criterion was applied during screening. The search strategy was developed collaboratively with a subject librarian. The MEDLINE strategy was then adapted to the syntax and fields of each platform. The three core concepts of interdisciplinary teamwork, falls prevention, and hospital setting were combined in every database, and a health-professional concept was added in MEDLINE, CINAHL, the Cochrane Library, and Europe PMC because the review question concerns healthcare professionals’ perspectives. Subject headings were combined with title and abstract terms in MEDLINE and CINAHL, whereas PsycINFO, the Cochrane Library, and Europe PMC were searched with free-text terms only. In Europe PMC, the teamwork and falls concepts were restricted to titles and abstracts, while the hospital and health-professional concepts were searched across all fields. In PsycINFO, an older-adult concept was used in place of the health-professional concept. This adaptation was intended to focus retrieval on the review population within a database whose scope extends well beyond hospital care. The involvement of healthcare professionals from two or more disciplines was instead assessed during screening against the eligibility criteria, as was the older-adult context in the other four databases. No language or publication-type limits were applied in any database, and these criteria were applied after deduplication. The full replicable search strategy for MEDLINE via EBSCOhost is presented in Table 1. The complete search strategies, platforms, search dates, limits, and retrieval totals for each database are reported in Supplementary Table S1. Third, the reference lists of all included studies were checked, and reports identified in this way were assessed against the same eligibility criteria. Finally, to test the sensitivity of the search, a supplementary search of MEDLINE via Ovid was conducted on 2 October 2026. This search omitted the health-professional concept, used the truncated term fall*, added terms for patient safety, safety culture, multifactorial interventions, and interprofessional education, and was limited to publications from 2013 to 2025, so it also covered the months outside the original MEDLINE date limits. The supplementary strategy is reported in Supplementary Table S1.

### 2.4. Study Selection

**Figure 1:**
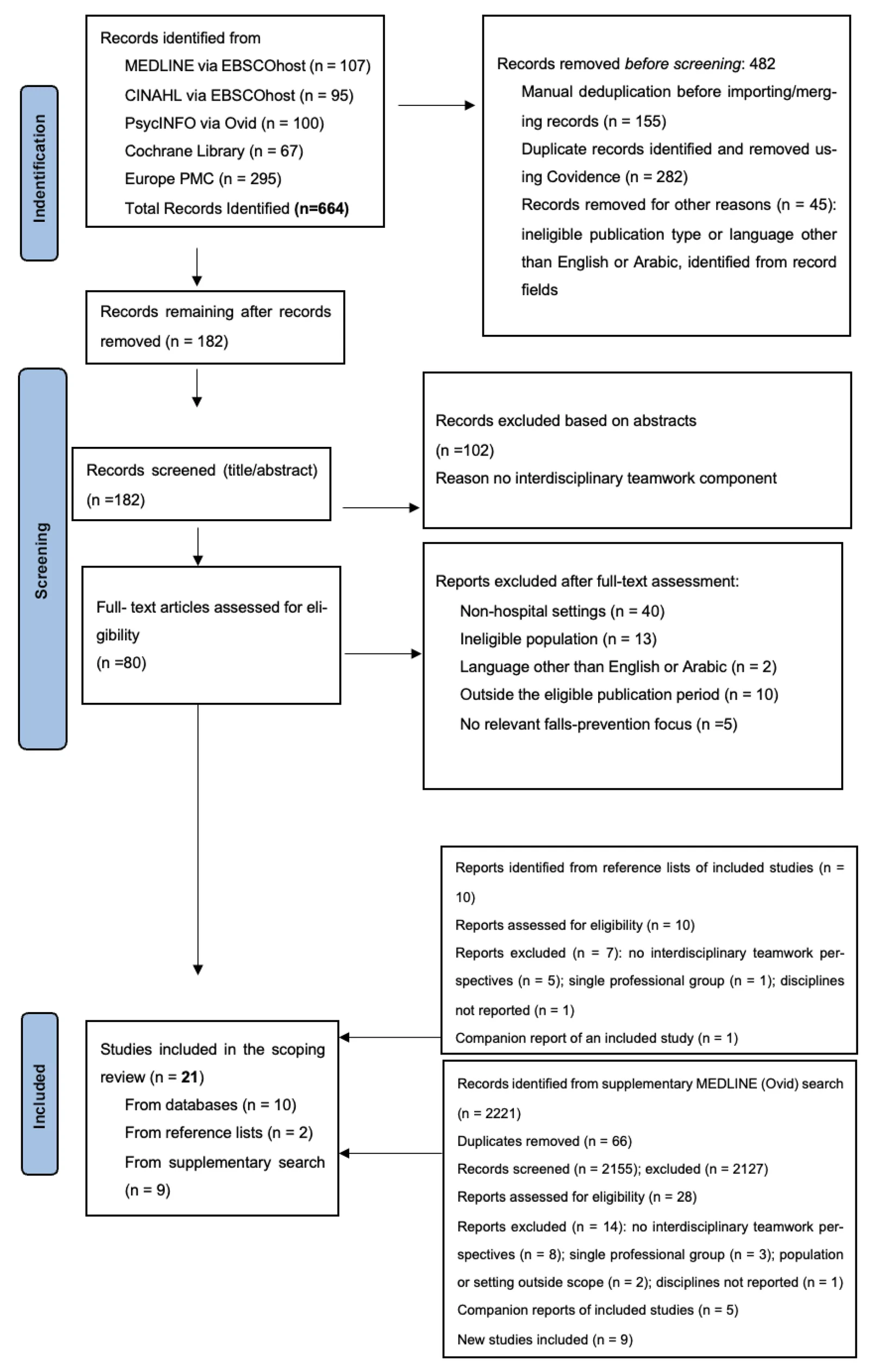
PRISMA-ScR flow diagram of study selection. The supplementary MEDLINE sensitivity search was conducted after the main search and screened separately, so its records are shown in a separate pathway.

Before screening, 482 records were removed in three sequential steps. First, 155 duplicate records were identified and removed manually before the records were imported into Covidence systematic review software. Second, after the remaining records from all five databases had been imported into Covidence, a further 282 duplicate records were identified and removed using Covidence. Third, after deduplication, 45 records were removed for other reasons, namely an ineligible publication type or publication in a language other than English or Arabic. These 45 records were identified from the publication-type and language fields of the bibliographic records rather than through title and abstract screening. Two reviewers (AA and GM) independently screened titles and abstracts. Full-text articles were then retrieved and independently assessed for eligibility by both reviewers. Reports identified through checking the reference lists of included studies were assessed independently by the same two reviewers against the same eligibility criteria, using the same process. Records retrieved by the supplementary search were imported into the same Covidence review, where duplicates of previously retrieved records were removed. Their titles and abstracts were screened in Covidence against the review eligibility criteria, and all reports identified as potentially eligible were assessed in full text independently by AA and GM. Screening decisions were checked against the previously included reports retrieved by the supplementary search, all of which were identified as eligible. The two reviewers’ decisions were compared at each stage, and all disagreements were resolved through discussion until consensus was reached, with a third reviewer (GP) consulted when consensus could not be reached. Study selection is reported using a PRISMA-ScR flow diagram (Figure 1).

### 2.5. Data Charting

Data were extracted using a customized version of the JBI data extraction tool [12]. The charting form was piloted on two studies by two reviewers (AA and GM) to ensure consistency and comprehensiveness before full extraction. Data were then extracted from all included studies, and one author (AA) checked all extracted data against the original articles. A second reviewer (GM) independently checked all extracted variables for every included study against the original articles to confirm accuracy and completeness. Any discrepancies were discussed and resolved by consensus, and the charted data were then discussed with the full review team. Extracted variables included author(s), year of publication, country, study aim, study design, setting, sample size, participant disciplines, analytical approach, and key findings relevant to interdisciplinary teamwork in falls prevention. In studies whose primary focus was the implementation of falls-prevention programs or falls education, only findings relating to interdisciplinary teamwork were charted. The charting process was iterative, allowing refinement as familiarity with the included studies increased, consistent with JBI recommendations [14]. A concise summary of included studies is presented in Table 2.

### 2.6. Data Analysis

Data from the included studies were analyzed using a descriptive analytical approach in accordance with the Arksey and O’Malley framework for scoping reviews [11]. Because the review included qualitative, quantitative, and mixed-methods studies, the analysis considered textual findings from qualitative components alongside reported outcomes and findings from quantitative components. Initial codes were generated from the extracted findings by AA, then discussed with GM and GP. During charting, each included study was also examined for whether it described the presence of several professional groups only or interdisciplinary processes such as shared information, coordinated role allocation, or joint decision-making, and this distinction was considered when interpreting the findings. In the four included studies that also reported patients’ or family members’ perspectives, these data were charted separately from those of healthcare professionals, used only where they related to interdisciplinary falls prevention, mainly in relation to patient engagement, and explicitly attributed to patients or family members when reported. The descriptive categories were not predetermined but emerged inductively from the extracted data of the included studies. Related codes were grouped into provisional categories, which AA and GM developed and refined iteratively as studies were compared to identify recurring patterns, trends, and concepts relevant to interdisciplinary teamwork in falls prevention. Disagreements about the categorization of findings were discussed and resolved by consensus. The contribution of each included study to each descriptive category is shown in Table 2. Quantitative findings were not pooled; instead, reported outcomes were charted descriptively within the relevant categories alongside qualitative findings. Where included studies reported effect estimates or confidence intervals, these were charted together with *p*-values, and no confidence intervals were estimated by the review team. Rather than undertaking a formal thematic synthesis, the analysis focused on organizing the extracted data into descriptive categories [16]. This approach allowed evidence to be integrated and mapped across diverse study designs while retaining the descriptive focus of a scoping review. Consistent with scoping review methodology, no formal critical appraisal of individual studies was undertaken [12,14]. Quantitative findings were therefore charted as reported by the authors of the included studies and were not interpreted as estimates of intervention effectiveness.

**Table 2:** Characteristics of included sources of evidence (*n* = 21).

Study	Country	Design/Analysis	Setting	Pop.	Participants, *n*; Disciplines	Study Aim	Key Findings Relevant to Interdisciplinary Teamwork	Evidence Type	Descriptive Categories
Albertini and Peduzzi (2024) [17]	Brazil	Qualitative case study; focus groups; content analysis (CIHC framework)	1 hospital (512 beds)	No age criterion	13; nursing, pharmacy, medicine, nutrition, PT, psychology, social work	To understand professionals’ perceptions and experiences of interprofessional falls-prevention practice	Limited awareness of other roles; separate records impeded information exchange; role clarification and co-responsibility were identified as essential	QL	C1, C2, C4
Hill et al. (2025) [18]	Australia	Qualitative; interviews and focus groups; directed content analysis (COM-B)	1 tertiary hospital (450 beds); 12 acute wards	No age criterion	40; nursing, PT, OT, physician	To identify barriers and enablers to implementing hospital falls-prevention education (SRP)	Appropriate delivery roles, repetition, patient readiness, carer involvement, time and resources shaped implementation; role allocation conditional on expertise and patient need	QL	C1, C3, C4
Heng et al. (2022) [19]	Australia	Qualitative; focus groups; thematic analysis	3 hospitals (1 acute, 2 rehabilitation)	No age criterion	23; nursing, PT, OT, allied health assistants	To explore barriers and enablers to delivering evidence-based patient falls-prevention education	Communication gaps, inconsistent documentation and role ambiguity constrained education; collaboration and collective responsibility were facilitators	QL	C1, C2, C3, C4
Ayton et al. (2017) [20]	Australia	Mixed methods; survey, focus groups, interviews; COM-B framework	24 acute wards, 6 hospitals	No age criterion	540; nursing, NUMs, DONs, QI, nursing educators	To identify barriers and enablers to implementation of the 6-PACK falls-prevention program	Falls prevention often viewed as nursing responsibility (80%); leadership, champions, training, resources and feedback were key enablers	QL, DQ (S)	C1, C3, C4
Randell et al. (2024) [21]	United Kingdom	Mixed-methods realist investigation; ethnography (251 h), interviews, record review	3 NHS trusts; 6 wards	≥65 or high-risk 50–64	81; nursing, allied health, pharmacy, medical, patients, carers	To determine how and in what contexts multifactorial falls-risk assessment and interventions are used in routine acute hospital practice	Nursing retained key responsibility for assessment and supervision; MDT meetings, huddles and visual tools supported but did not ensure shared responsibility	QL	C1, C2, C3
McLennan et al. (2024) [22]	Australia	Qualitative; interviews and focus groups; inductive thematic analysis	2 acute public hospitals	No age criterion	50; nursing, PT, OT, medical officers, managers, patients, family members	To explore multiple stakeholder perspectives on implementing fall-prevention programs in acute hospitals	Stakeholders located responsibility elsewhere; disempowerment and competing priorities constrained implementation; shared responsibility and patient/family involvement proposed as enablers	QL	C1, C3, C4
Jones et al. (2019) [23]	United States	Quasi-experimental; longitudinal and cross-sectional; AHRQ HSOPSC survey	16 small rural hospitals	No age criterion	814 teamwork survey and 1843 safety-culture survey respondents; nursing, pharmacy, PT/OT, QI, administration	To evaluate associations between post-fall huddles, repeat-fall rates and safety-culture perceptions	Huddle participation associated with more positive teamwork and safety-culture perceptions; moderate inverse correlation with repeat falls that was not statistically significant (r = −0.47, *p* = 0.07); observational design; no confidence intervals reported	ST (F, S)	C1, C2, C3, C4
Hill et al. (2016) [24]	Australia	Qualitative; focus groups; thematic analysis	5 rehabilitation wards	Aged-care rehabilitation	30; nursing, allied health, medical, QI	To examine staff response to an individualized patient falls-prevention education program (SRP)	Patient education created a positive falls-prevention culture; staff, patients and educator worked as a team; patient empowerment reinforced staff practice	QL	C1, C2, C4
Lopez-Jeng and Eberth (2020) [25]	United States	Pre–post evaluation; AHRQ HSOPSC survey; Five As behavior-change model	1 critical-access hospital	No age criterion	78 pre-test and 40 post-test respondents; nursing, pharmacy, PT/OT/speech, administration, physicians, dietitians	To evaluate a four-week interdisciplinary falls-prevention education program on safety culture	Significant increases in percent positive scores for management support (+12.0 points), patient safety perceptions (+12.8 points), teamwork across units (+11.6 points) and handoffs (+13.0 points); no confidence intervals reported	PP, ST (S)	C1, C2, C3, C4
Shaw et al. (2023) [26]; companion report Heng et al. (2022) [27]	Australia	Mixed methods; pre–post surveys and interviews; independent samples t-tests, thematic analysis. Companion report: mixed-methods trial with patient surveys and the same staff interviews [27]	1 private acute hospital	No age criterion	34 pre-test and 25 post-test respondents; 7 interviewees; nursing, PT, OT, management	To design, deliver and evaluate an IPE program preparing staff to implement the SRP, and to identify barriers and enablers to implementing the program [27]	One-hour IPE did not change collaboration scores; participants sought preparation, interactive practice, cognitive aids and refresher support. Staff described limited collaboration on falls education during the trial; senior staff support and consistent messages were enablers [27]	QL, PP, ST (S)	C2, C3, C4
Milisen et al. (2013) [28]	Belgium	Descriptive multicenter feasibility study; questionnaire after a one-month guideline test; descriptive statistics	17 geriatric wards in 17 hospitals	Geriatric wards	49; geriatricians, nurses, PT, OT	To determine the feasibility of implementing a multidisciplinary fall prevention guideline on geriatric wards	Several disciplines felt responsible and capable for most assessments and interventions; barriers included lack of communication between disciplines (35.8%), insufficient multidisciplinary teamwork (28.3%) and uncertainty about responsibility	DQ (S)	C1, C2, C3
Porter et al. (2018) [29]	United States	Qualitative; focus groups and interviews; framework analysis	1 academic medical center (739 beds)	No age criterion	20; nursing, nurse leaders, pharmacy, medicine, PT, physician assistants	To explore interprofessional team perspectives on sustaining an evidence-based fall prevention program	Non-nurse clinicians were largely unaware of nursing fall-risk tools and cues; most placed primary responsibility on nurses while recognizing that coordinated interprofessional teamwork was needed	QL	C1, C2, C3
Antonacci et al. (2023) [30]	United Kingdom	Qualitative; interviews; constant comparative analysis	7 acute hospitals	Over 65 years (audit)	18; consultants, matrons, nursing, PT	To explore how front-line staff use national falls audit feedback to improve practice	Ownership, clear responsibilities, leadership and communication across professional groups enabled improvement; staffing, turnover and falls seen as an older-people’s-ward problem were barriers	QL	C1, C2, C3
Macchiavello et al. (2025) [31]	United Kingdom	QI collaborative; learning sessions, audits, participant survey	5 older adults’ mental health wards	Older adults, mental health	27–30 per session; nursing, OT, PT, pharmacy, medicine, dietetics, managers	To reduce falls through a multidisciplinary QI collaborative	Multidisciplinary participation supported shared learning; engaging medical staff and staffing pressures were challenges; falls unchanged overall	DQ (S, F)	C1, C3, C4
McLennan et al. (2025) [32]	Australia	Mixed methods; surveys, interviews; content analysis (i-PARIHS)	4 acute wards, 1 hospital	No age criterion	99 survey responses (mostly nursing), 12 interviews; nursing, PT, dietetics	To assess QI education and facilitation for tailored multicomponent falls prevention	Unclear team roles, lack of accountability, competing priorities and staffing were barriers; empowered decision-making and leadership were facilitators	QL, DQ (S)	C1, C2, C3, C4
Ploeg et al. (2018) [33]	Canada	Mixed methods; interviews, focus groups, fall rates; template analysis	3 acute hospitals	Older adults	82; RN, RPN, OT, PT, managers, educators, leaders	To assess a mentored, sustainability-focused falls guideline implementation	Interprofessional teamwork, a whole-team approach, leadership and champions were valued; no significant reduction in fall rates	QL, ST (F)	C1, C3, C4
Rababah et al. (2024) [34]	Jordan	Qualitative; reflective journals; thematic analysis	2 neurology units, 2 hospitals	No age criterion	23; physicians, nursing, pharmacy, PT, OT	To explore insights on an interdisciplinary falls prevention program (STEADI)	The interdisciplinary approach encouraged shared decision-making, communication and role awareness; time and space constraints limited use	QL	C1, C2, C3, C4
Spiva et al. (2014) [35]	United States	Quasi-experimental with comparison group; questionnaires, observations	Orthopedic and neurology units, 2 hospitals	No age criterion	34; nursing, pharmacy, PT, physicians	To evaluate team training on teamwork, safety culture and falls	Communication about error and observed interprofessional communication improved; perceptions of current teamwork decreased, attributed to greater awareness; no significant differences between groups or in falls	ST (S, F)	C2, C4
Stuby et al. (2025) [36]	Switzerland	Mixed methods; survey, interviews	General internal medicine wards, 2 teaching hospitals	Older adults	Survey and 25 interviews; nursing, resident physicians	To assess the feasibility and acceptability of a QI intervention for fall risk assessment	Feasible and accepted; a quarter of clinicians reported an impact on interprofessional collaboration; healthcare assistants were not included in rounds; workload was a barrier	QL, DQ (S)	C1, C2, C3, C4
Toye et al. (2019) [37]	Australia	Mixed methods; audits, surveys, focus groups	4 medical wards, 1 tertiary hospital	≥70 years	105 survey responses, 21 focus-group participants; nursing, medical, allied health	To examine bed moves, ward environment and staff perspectives on falls	Interprofessional collaboration was rated good; poor communication between wards during bed moves and time pressures were seen to increase falls risk	QL, DQ (S)	C2, C3
Wynaden et al. (2016) [38]	Australia	Qualitative; focus groups; content analysis	Older adult mental health units	Older adults, mental health	28; nursing, PT, OT	To understand falls risk among older adult mental health inpatients	Generic tools were unsuitable; collaborative multidisciplinary assessment was fundamental, with each discipline contributing distinct skills	QL	C1, C2

Note. Pop. = patient population as reported by each source, where No age criterion indicates that no numerical patient age criterion was reported; Evidence type = type of evidence underlying the key findings charted for each source, where QL = qualitative findings, DQ = descriptive quantitative findings, PP = uncontrolled pre-post findings, ST = statistically tested association or difference, F = outcome directly related to falls, and S = surrogate outcome such as teamwork, safety-culture, or role perceptions; Descriptive Categories = descriptive categories in which findings from each source are reported, where C1 = interprofessional roles, responsibilities and shared accountability, C2 = communication and coordination, C3 = organizational and systemic influences, and C4 = interprofessional education and patient engagement. Heng et al. [27] is a companion report of Shaw et al. [26], and both are charted in the same row. PT = physiotherapy; OT = occupational therapy; QI = quality improvement; NUMs = Nurse Unit Managers; DONs = Directors of Nursing; IPE = interprofessional education; SRP = Safe Recovery Programme; MDT = multidisciplinary team; CIHC = Canadian Interprofessional Health Collaborative; AHRQ HSOPSC = Agency for Healthcare Research and Quality Hospital Survey on Patient Safety Culture; COM-B = capability, opportunity, motivation, and behavior model; NHS = National Health Service; i-PARIHS = integrated Promoting Action on Research Implementation in Health Services framework; STEADI = Stopping Elderly Accidents, Deaths, and Injuries; RN = registered nurse; RPN = registered practical nurse. Participant numbers are reported as presented in each source and may include survey respondents, interview/focus-group participants, or other stakeholders.

## 3. Results

### 3.1. Study Selection

A total of 664 records were identified through database searching: MEDLINE (*n* = 107), CINAHL (*n* = 95), PsycINFO (*n* = 100), the Cochrane Library (*n* = 67), and Europe PMC (*n* = 295). Before screening, 482 records were removed. In sequence, 155 duplicates were removed manually before the records were imported into Covidence, 282 duplicates were identified and removed using Covidence after all records had been imported, and 45 records were then removed because of an ineligible publication type or a language other than English or Arabic, as identified from bibliographic record fields. The remaining 182 records were screened by title and abstract, and 102 were excluded because they did not include an interdisciplinary teamwork component. Eighty full-text articles were assessed for eligibility. Of these, 70 were excluded: 40 concerned non-hospital settings, 13 had an ineligible population, 10 were published outside the eligibility period, 2 were published in a language other than English or Arabic, and 5 did not have a relevant falls-prevention focus. Ten studies identified through database searching met the eligibility criteria. Checking the reference lists of these studies identified 10 further reports, none of which had been retrieved by the database searches. Of these, 2 were eligible new studies, 1 was a companion report of an already included study and was charted with that study, and 7 were excluded because they did not examine professionals’ perspectives on interdisciplinary teamwork (*n* = 5), involved a single professional group (*n* = 1), or did not report the professional disciplines of participants (*n* = 1). The supplementary MEDLINE search retrieved 2221 records, of which 66 were duplicates of previously retrieved records, and the remaining 2155 records were screened by title and abstract. Of these, 2127 were excluded, and 28 reports were assessed in full text. Five were companion reports of already included studies and were not charted separately, and 14 were excluded because they did not examine professionals’ perspectives on interdisciplinary teamwork (*n* = 8), involved a single professional group (*n* = 3), concerned a population or setting outside the scope of the review (*n* = 2), or did not report the professional disciplines of participants (*n* = 1). The remaining 9 reports were eligible new studies. The supplementary search also retrieved the three reports previously identified through reference lists. In total, 21 studies were included in the scoping review, 10 from database searching, 2 from reference lists, and 9 from the supplementary search. The study selection process is presented in the PRISMA-ScR flow diagram (Figure 1).

### 3.2. Study Characteristics

The 21 included studies were published between 2013 and 2025 and used qualitative, mixed-methods, and quantitative designs. Most explored healthcare professionals’ experiences, perceptions, barriers, and enablers of interdisciplinary falls-prevention practice. Five statistically tested associations or changes in teamwork-, safety-, or falls-related outcomes, and six reported descriptive survey data, as indicated by the evidence type codes in Table 2. One study was reported in two publications, which were charted together in Table 2. The design, sample, instruments, outcomes, main results, and principal methodological limitations of the eight studies reporting quantitative findings relevant to interdisciplinary teamwork are summarized in Supplementary Table S2. Studies were conducted in hospital inpatient settings across eight countries, with nine undertaken in Australia, four in the United States, three in the United Kingdom, and one each in Belgium, Brazil, Canada, Jordan, and Switzerland. Participants included nurses, allied health professionals, physicians, physician assistants, pharmacists, managers, educators, quality-improvement staff, support workers, and, in four studies, patients or family members. Sample sizes and settings varied substantially, from a single-site qualitative study to a multicenter pre-implementation study. Table 2 summarizes the characteristics and findings of each included source and the type of evidence underlying its key findings. Four studies reported outcomes directly related to falls, none of which reported a statistically significant reduction in falls, and all other quantitative findings concerned surrogate outcomes such as teamwork or safety-culture perceptions.

### 3.3. Descriptive Synthesis of Results

The findings were organized into four descriptive categories, namely (1) Interprofessional Roles, Responsibilities, and Shared Accountability, (2) Communication and Coordination in Falls Prevention, (3) Organizational and Systemic Influences on Interdisciplinary Teamwork, and (4) Interprofessional Education and Patient Engagement in Falls Prevention.

#### 3.3.1. Interprofessional Roles, Responsibilities, and Shared Accountability

Role clarity emerged as an important condition for interdisciplinary teamwork in falls prevention, yet it was inconsistently achieved across the included studies. Health professionals were generally aware of their own contributions to falls prevention but demonstrated limited understanding of other team members’ roles and responsibilities [17]. In some settings, specific professional groups were identified as better suited to deliver falls prevention education, particularly physiotherapists and occupational therapists, although participants acknowledged that any allied health professional could do so with appropriate guidance [18]. In older adults’ mental health units, nurses, occupational therapists, and physiotherapists described distinct but complementary contributions to falls-risk assessment, with nurses attending to comorbidities and medications, occupational therapists to cognition, and physiotherapists to gait and mobility aids [38]. In neurology units, interdisciplinary training was reported to increase professionals’ awareness of one another’s roles in falls prevention [34]. However, role boundaries were frequently blurred. In one study, two nurses held the view that formal falls education was the role of allied health professionals [19], and in another, nurses suggested that falls-prevention messages to patients would be more effective coming from physiotherapists or doctors [22]. In a Belgian multicenter study, several disciplines considered themselves responsible and capable of most fall-risk assessments and interventions [28]. Nevertheless, uncertainty about which discipline was responsible for particular tasks, unclear team member roles, and a lack of accountability were described as barriers to implementing falls-prevention interventions [28,32]. These findings suggest that professional identity and disciplinary boundaries continue to shape how falls prevention responsibilities are distributed within the team.

Several included studies described falls prevention as nursing-centered despite stated commitments to multidisciplinary approaches. Patient supervision, risk assessment, and routine falls-prevention activities were commonly described as nursing responsibilities, which could constrain wider shared accountability [20,21]. In one multisite pre-implementation study [20], 80% of nurses believed they were responsible for falls prevention, and senior staff agreed that nurses were primarily responsible while recognizing the value of multidisciplinary input. Similar tensions around role allocation were reported in Australian, Brazilian, Swiss, UK, and US settings [17,21,22,29,30,32,36]. In a US academic medical center, most participants identified bedside nurses as primarily responsible for fall-risk assessment and prevention, although many recognized that coordinated interprofessional teamwork was needed for prevention strategies to succeed [29]. In English and Welsh hospitals, staff described falls prevention as being regarded as a concern of older people’s wards, and participants emphasized the need for ownership and clear responsibilities across departments [30]. In Swiss medical wards, healthcare assistants were described as having frequent contact with patients but as not taking part in medical rounds or nurse–physician discussions about falls risk [36]. These findings indicate a recurring pattern in the mapped literature but do not demonstrate that nursing-centered responsibility operates uniformly across hospital contexts.

Despite the prevailing pattern of nursing-centered responsibility, a strong aspiration for collective ownership of falls prevention was evident across the evidence base. Participants in two studies, including patients and family members in one [22], expressed optimism about shared responsibility across disciplines, organizational levels, and between staff and patients [20,22]. Structured interdisciplinary processes appeared to support this aspiration in practice. In one study, each participating hospital established an interprofessional coordinating team, with members from nursing, pharmacy, physical or occupational therapy, and quality improvement, to lead the implementation of fall-risk-reduction practices, including post-fall huddles [23]. Similarly, collaborative education programs facilitated a sense of teamwork between disciplines and between staff and patients, with one study describing how the program created a shared culture around falls prevention in which different professional groups reinforced each other’s contributions [24]. Staff in three Canadian hospitals described a whole-team approach to falls prevention, supported by champions and leaders, as important for sustaining a falls-prevention guideline [33], and in older adults’ mental health wards, multidisciplinary participation in a quality-improvement collaborative was considered central, although engaging medical staff was challenging [31]. Senior staff consistently articulated the view that falls prevention should be “everyone’s responsibility” [20], and professionals from seven disciplines in one hospital, including nurses and nursing technicians, identified shared accountability as essential for effective falls prevention [17]. However, structured processes for putting this aspiration into practice were described less often than the aspiration itself. Structured interdisciplinary processes, including collaborative education, post-fall huddles, interdisciplinary health education, interdisciplinary training workshops, and multidisciplinary quality-improvement collaboratives, were described in several studies [23,24,25,31,32,33,34]. The gap between the widely expressed commitment to shared accountability and the more limited description of its implementation was a recurring pattern in the mapped studies. Because this review mapped perceptions, the limited description of implementation does not show that structured interdisciplinary processes were absent in practice.

#### 3.3.2. Communication and Coordination in Falls Prevention

Effective communication was consistently described as an important means through which interdisciplinary coordination was supported in hospital falls prevention. When communication processes were well established, they facilitated shared understanding, timely information exchange, and coordinated care across professional groups [23,24,26,34,38]. Collaborative education programs were described as creating mutually reinforcing communication between disciplines, where different professional groups reinforced each other’s contributions to falls prevention and where staff, patients, and educators worked together toward shared safety goals [24]. Structured post-fall huddles similarly facilitated collective sensemaking across professional boundaries, providing a forum for staff from nursing, pharmacy, therapy, and quality improvement to identify the causes of a fall and develop a coordinated plan to prevent recurrence [23]. Shared interprofessional education also reinforced existing communication between nurses and allied health professionals, with participants reporting that training together placed them “on the same page” when implementing falls prevention strategies [26]. However, in a companion report of the same study, staff described limited collaboration specifically on falls education during the trial, despite reporting strong interprofessional teamwork beforehand [27]. In hospitals participating in a national falls audit, feedback was shared in written and verbal formats with different professional groups, and open discussion of the findings was valued [30]. On one acute ward, the local team chose to refine interdisciplinary communication about patients’ mobility status as part of its falls-prevention work [32]. In Swiss medical wards, about a quarter of clinicians reported that an intervention to systematize fall risk assessment had affected interprofessional collaboration, possibly because collaboration between nurses and residents was already established [36]. These findings suggest that communication was more than a supporting factor in the included studies and appeared to shape how interdisciplinary teamwork functioned in practice.

However, communication breakdowns were equally prominent across the evidence base, and the barriers described were mainly related to systems and workflow. Documentation discrepancies between professional groups emerged as a recurring barrier, with allied health professionals and nurses maintaining separate records that were not routinely shared or accessible to other team members [17,19]. In one hospital, dual electronic medical record systems meant that medical staff could not access notes written by pharmacists, nutritionists, or physiotherapists without navigating a separate system, reducing the time available for direct patient care [17]. Beyond documentation, verbal communication about falls prevention occurred both formally through multidisciplinary team meetings and informally through corridor conversations, but was not systematically structured across disciplines in most settings [21]. Handover periods and transfers between wards were identified as particularly vulnerable points, where critical patient information needed to transfer between shifts, wards, and professional groups but was frequently lost or incompletely communicated [19,37]. Allied health professionals reported that their mobility recommendations were sometimes overlooked or disregarded by other staff, indicating that even when communication occurred, it did not always translate into coordinated action [19]. In another study, non-nurse clinicians were largely unaware of the fall-risk scale and visual cues that nurses used to communicate fall risk, and relied instead on their own assessments or on face-to-face communication [29]. In a Belgian multicenter study, 35.8% of healthcare workers identified a lack of communication between disciplines, and 28.3% identified insufficient multidisciplinary teamwork, as barriers to implementing a multidisciplinary fall prevention guideline [28]. The mapped studies described communication barriers in falls prevention mainly in relation to institutional information systems and workflow structures. Individual attitudes to collaboration were not examined directly, so their contribution cannot be judged from this evidence.

Three studies examined structured communication interventions designed to address these systemic barriers, and the available quantitative evidence was concentrated in this area. In one study of post-fall huddles implemented with pocket guides and standardized documentation forms, staff who had participated in huddles reported more positive perceptions of teamwork across hospital departments than staff who had not (75% versus 66% positive, *p* = 0.011). Huddle participants also responded more positively to three of four handoff and transition items, although the difference for the handoffs and transitions dimension as a whole did not reach statistical significance (61% versus 52% positive, *p* = 0.07). These findings suggest that formalizing interdisciplinary communication around fall events may strengthen cross-departmental coordination [23]. Similarly, an interdisciplinary education program that included dedicated sessions on teamwork and communication was followed by higher percent positive scores for teamwork across units (from 65.9% to 77.5%, a difference of 11.6 percentage points, *p* = 0.010) and handoffs and transitions (from 43.9% to 56.9%, a difference of 13.0 percentage points, *p* = 0.008) [25]. Neither study reported confidence intervals, and the education program compared separate anonymous convenience samples of 78 pre-test and 40 post-test respondents [23,25]. In a third study, team training based on Team Strategies and Tools to Enhance Performance and Patient Safety (TeamSTEPPS), evaluated against a comparison group, was followed in the intervention group by improvement in feedback and communication about error (*p* < 0.01) and in observed interprofessional communication. Perceptions of the current state of teamwork decreased over time, which the authors attributed to greater awareness of the gap between best practice and day-to-day teamwork; differences between the groups were not statistically significant, and the 62% reduction in falls was not statistically significant (*p* = 0.15) [35]. These interventions shared a common feature in that they created structured opportunities for professionals from different disciplines to discuss falls prevention together, rather than relying on informal or incidental communication. However, the evidence for structured communication tools was limited to these three quantitative studies, and none established a causal relationship between the communication intervention and actual falls reduction. While the available evidence suggests that formalizing interdisciplinary communication processes may improve teamwork perceptions and safety culture, the relationship between improved communication and measurable falls outcomes remains largely untested.

#### 3.3.3. Organizational and Systemic Influences on Interdisciplinary Teamwork

Organizational and systemic factors shaped the conditions within which interdisciplinary teamwork could operate, and resource constraints were the most consistently reported barrier across the evidence base. Heavy workloads, insufficient staffing, and competing clinical priorities were described as limiting the time and opportunity available for interprofessional communication and collaboration [18,19,20,22,27,29,30,31,32,34,36,37]. Falls prevention competed with other clinical demands for staff attention, and even professionals who valued interdisciplinary approaches acknowledged that time pressures often prevented them from engaging meaningfully with colleagues from other disciplines [22]. In practical terms, insufficient falls prevention equipment and the absence of tracking systems for resources across wards meant that even when staff identified a patient as high risk, they did not always have access to the interventions needed to act on that assessment [20]. In a US academic medical center, staffing levels, unit layouts, and practices regarding patient room doors were described as affecting fall prevention, and non-nurse clinicians were often unaware of unit-level falls data [29]. In a Belgian multicenter study, the time required to apply a multidisciplinary guideline was the most frequently reported barrier (81.1%), and 15.4% of respondents reported a lack of interest from hospital management [28]. In hospitals using national falls audit data, low organizational priority for falls and limited quality-improvement skills were also described as barriers [30], and in Swiss medical wards, larger teams were described as harder to coordinate [36]. Only 32% of nurses in one multisite study reported receiving useful training from falls prevention leaders [20], suggesting that professional development for interdisciplinary falls prevention was not consistently prioritized within organizational resource allocation. These constraints indicate that individual willingness to collaborate may be insufficient when organizational structures do not create the opportunity for interdisciplinary interaction.

Leadership emerged as a critical enabler of interdisciplinary teamwork for falls prevention, operating at both the ward and organizational levels. Staff who participated in structured interdisciplinary activities reported significantly more positive perceptions of team leadership than those who did not, and this extended across multiple leadership behaviors, including providing opportunities for post-fall discussion, resolving conflicts, and ensuring awareness of changes affecting falls prevention [23]. At the organizational level, interdisciplinary education led by a hospital-wide falls prevention committee was associated with a significant increase in management support for patient safety (from 70.5% to 82.5% positive, a difference of 12.0 percentage points, *p* = 0.014), suggesting that visible organizational commitment can reinforce the conditions for interdisciplinary teamwork [25]. Ward-level champions were identified as particularly effective enablers, serving as bridges between frontline staff, management, and falls committees by providing education, support, and ongoing accountability for falls prevention practice [20,33]. Support and reminders from senior staff were also described as enablers in one study, and participants recommended greater engagement of leaders [27]. Leadership and management support were similarly described as enablers in studies of audit-based improvement, guideline implementation, and ward-level falls-prevention projects [30,32,33]. However, leadership structures were not always aligned with interdisciplinary goals. In one study, leadership for falls prevention resided with senior nurses while falls link practitioners lacked the authority to allocate resources or influence practice beyond their immediate ward [21]. This disconnect between designated leadership roles and actual decision-making authority suggests that effective falls prevention leadership requires both formal organizational endorsement and practical authority distributed across disciplines rather than concentrated within a single professional group.

Organizational safety culture emerged as both an influence on and an outcome of interdisciplinary teamwork for falls prevention. Structured interdisciplinary activities were associated with more positive perceptions across multiple safety culture domains, including organizational learning, non-punitive response to error, and teamwork across departments (75% versus 66% positive, *p* = 0.011) [23]. An interdisciplinary education program was followed by similar improvements, with overall perceptions of patient safety increasing by 12.8 percentage points (from 65.1% to 77.9% positive, *p* = 0.005) and teamwork across units by 11.6 percentage points (*p* = 0.010) following four weeks of collaborative education [25]. Neither study reported confidence intervals for these estimates. These results should be interpreted cautiously because the huddle study compared self-selected participants with non-participants at a single time point [23], and the education program had no control group and was evaluated during a period in which the hospital introduced a new falls-reporting system [25]. Both studies also reported multiple comparisons across survey items and dimensions without adjustment for multiple testing, so individual *p*-values should be read with caution. These quantitative findings suggest that when interdisciplinary processes are embedded into routine practice, they may positively influence the wider organizational culture around falls prevention. However, organizational commitment was not consistently evident across settings. Staff in one multisite study described falls risk assessment as a routine compliance activity, noting that assessment tools were often completed through habitual “tick-box” responses rather than through genuine clinical reasoning about individual patient risk [20]. This disconnect between assessment processes and meaningful interdisciplinary engagement was reinforced by the finding that nearly a quarter of nurses were undecided about whether falls could be prevented at all [20], suggesting that safety culture may be undermined when staff perceive falls as inevitable rather than preventable. Taken together, these findings indicate that safety culture in hospital falls prevention is not a static organizational attribute but may be shaped by how interdisciplinary teamwork is structured, supported, and sustained over time.

#### 3.3.4. Interprofessional Education and Patient Engagement in Falls Prevention

This category brought together interprofessional education and patient engagement because the two emerged from the included studies as closely linked. Four of the studies contributing to this category examined patient engagement in the context of patient falls-prevention education programs [18,19,24,26], and three of these studies concerned the Safe Recovery Programme [18,24,26]. Interprofessional education was widely recognized as a potential means of building interdisciplinary capability in falls prevention, but the available evidence suggested that a single education session was insufficient to produce measurable changes in collaborative practice. A single 1 h interprofessional education session did not result in statistically significant changes in interprofessional collaboration scores across any of the 20 items measured, which the authors suggested may reflect already high pre-education ratings [26]. Similarly, only 32% of nurses in one multisite study felt they received useful training from falls prevention leaders [20], and participants across several studies expressed a preference for face-to-face, case-study-based education with ongoing refresher sessions rather than one-off training events [18,20]. In contrast, sustained and structured interdisciplinary activities were associated with more positive perceptions in two studies, although this evidence was limited. Post-fall huddles conducted over a two-year period were associated with more positive teamwork perceptions, and huddle participation showed a moderate inverse correlation with repeat fall rates. However, this correlation did not reach statistical significance (r = −0.47, *p* = 0.07) and should not be interpreted as evidence that huddles reduced repeat falls [23]. In a four-week interdisciplinary education program, participants who attended all four sessions had slightly higher post-session knowledge scores than those who attended fewer sessions, although these comparisons were descriptive and no pre-session knowledge data were collected [25]. Other studies used interdisciplinary workshops with case discussions and hands-on tasks [34], team training based on TeamSTEPPS [35], e-learning with reminder quizzes [36], multidisciplinary learning sessions within a quality-improvement collaborative [31], education and networking sessions supporting guideline implementation [33], and quality-improvement education with clinical facilitation for ward teams [32]. Delivery was not always straightforward, as e-learning completion differed markedly between two hospitals (86% versus 45%) [36], and team-training sessions did not always include an interdisciplinary group of participants [35]. Collaborative education programs involving the full ward team were described as facilitating a positive culture around falls prevention in which staff, patients, and the educator worked together [24]. Education and teamwork activities in the included studies varied in duration, frequency, and interprofessional composition, but the studies did not isolate the effects of these features, so their relative importance remains uncertain.

Patient engagement in falls prevention was consistently described as an activity that depended on interdisciplinary coordination rather than individual professional effort. Patients who received collaborative education from multiple disciplines became more proactive in their own falls prevention, with staff observing that patients were more engaged when different professional groups reinforced consistent safety messages [24]. Staff in a companion report of a hospital education trial similarly described fostering patient empowerment and consistent reinforcement of falls messages across and within professions as enablers of implementation [27]. More than two-thirds of participants in one study recommended empowering patients through individualized education; participants also identified family involvement as a mechanism for reinforcing education [19]. Staff in another study described new opportunities to collaborate with patients and families when implementing a falls-prevention guideline [33], and on one acute ward, staff delivery of oral patient education about fall risk was selected as a local intervention [32]. However, patient engagement was constrained by clinical and contextual factors that varied across settings and across the course of a hospital admission. Healthcare professionals described cognitive impairment, language barriers, and acute illness as limiting patients’ capacity to participate meaningfully in falls prevention [18,22]. Effective patient communication required active listening and rapport, and professionals in one study noted that patients frequently signed awareness documents without absorbing their content, suggesting that information provision alone was not sufficient to achieve genuine engagement [17]. Patients themselves reported feeling unable to prioritize falls prevention when overwhelmed by pain, medication effects, and the unfamiliar hospital environment, particularly early in their admission [22]. These findings indicate that patient engagement in hospital falls prevention is not a standalone educational activity but an interdisciplinary endeavor requiring coordinated input from multiple professional groups, adapted to each patient’s clinical status and readiness to participate.

## 4. Discussion

This scoping review mapped the existing literature on healthcare professionals’ perspectives on interdisciplinary teamwork for falls prevention among older adults in hospital settings. Twenty-one studies published between 2013 and 2025 across eight countries were identified, employing qualitative, quantitative, and mixed-methods designs. With the addition of studies from Canada, Jordan, and Switzerland, most findings appeared across several health systems, although the evidence remained weighted toward Australia, where nine of the 21 studies were conducted. Findings on professional roles and responsibilities were reported in all eight countries [17,18,19,20,21,22,23,24,25,28,29,30,31,32,33,34,36,38], and role ambiguity or a nursing-centered distribution of falls-prevention work was described in Australian, Belgian, Brazilian, Swiss, UK, and US hospitals [17,19,20,21,22,28,29,30,32,36]. Communication and coordination findings came from seven countries [17,19,21,23,24,25,26,27,28,29,30,32,34,35,36,37,38], although structured communication activities such as post-fall huddles, interdisciplinary education, and team training were evaluated statistically only in US and Australian hospitals [23,25,26,35]. Organizational influences, including workload, staffing, leadership, and ward champions, were also described in seven countries [18,19,20,21,22,23,25,26,27,28,29,30,31,32,33,34,36,37], which suggests that these constraints were not specific to any single health system. Evidence on interprofessional education was similarly drawn from several countries [18,20,24,25,26,31,32,33,34,35,36], whereas evidence on patient engagement came mainly from Australian studies [18,19,22,24,26,27,32], several of them linked to the Safe Recovery Programme [18,24,26], with additional findings from Brazil and Canada [17,33]. Findings on patient engagement may therefore be particularly shaped by Australian hospital systems. Only one included study was conducted in the Middle East [34], and it was published in English, so no Arabic-language study was identified despite the eligibility of Arabic-language publications. This may reflect limited published research on interdisciplinary teamwork for hospital falls prevention in the region, the absence of an Arabic-language database from the search, or both, and it identifies a priority for future research. Across the mapped studies, a recurring pattern was that healthcare professionals viewed interdisciplinary teamwork as important for falls prevention, while its translation into structured collaborative practice was variable. In all four descriptive categories, the gap between professionals’ views of teamwork and their day-to-day experience of collaborative practice was a prominent feature of the evidence base. This aspiration-implementation gap mirrors a broader pattern observed in the interprofessional collaboration literature, where a Cochrane review concluded that although interprofessional collaboration interventions can improve healthcare processes, the evidence for their impact on patient outcomes remains limited and heterogeneous [9]. The small and predominantly descriptive nature of the evidence mapped by this review suggests that despite widespread policy endorsement of interdisciplinary approaches to falls prevention, the empirical investigation of how these approaches operate in hospital settings remains at an early stage.

Although the descriptive categories were developed inductively, interpreting them against the World Health Organization Framework for Action on Interprofessional Education and Collaborative Practice [8] and the TeamSTEPPS model [39] provided both conceptual and practical insights into how interdisciplinary teamwork was described in the mapped studies. TeamSTEPPS describes teamwork through five domains, namely team structure, leadership, communication, situation monitoring, and mutual support [39]. The first descriptive category, roles and shared accountability, corresponded mainly to team structure, and the second, communication and coordination, corresponded to communication and situation monitoring, including handovers and post-fall huddles. The third category corresponded to leadership and to the institutional, working-culture, and environmental mechanisms that the World Health Organization framework identifies as shaping collaborative practice [8]. The fourth reflected that framework’s emphasis on interprofessional education as a route to collaborative practice and the place of patients within the team [8,39]. Mutual support did not emerge as a distinct finding in the mapped studies and was measured only as one dimension of TeamSTEPPS teamwork questionnaires in two studies [23,35]. Interpreted in this way, communication appeared to be one component of teamwork rather than a synonym for it, and safety culture appeared to form part of the organizational context in which teamwork operates rather than a teamwork process in itself. The included studies rarely made these distinctions, and future research would benefit from examining teamwork, communication, and safety culture separately.

The mapped studies suggest that shared accountability remains incompletely operationalized in hospital falls prevention. Professionals often understood their own contributions more clearly than those of other team members, while nursing retained a substantial share of routine falls-prevention work [17,21,22,29]. One possible interpretation, not tested in the included studies, is that this pattern partly reflects how professional roles, scopes of practice, and nurse staffing are organized in the health systems that contributed most of the evidence, and it may differ where allied health or medical staff hold greater responsibility for falls prevention. Explicit role clarification and clearer definitions of interdisciplinary teamwork may help future studies distinguish teamwork from related constructs such as communication and safety culture. Two studies extended this evidence to older adults’ mental health wards, where staff described generic falls-risk tools as unsuitable for this population and multidisciplinary assessment and participation as essential [31,38]. Because evidence on falls prevention in these wards remains limited [31], this setting warrants further research.

The mapped studies described communication barriers as often embedded in information systems and workflow practices. Dual electronic medical records, inconsistent documentation, and unstructured handovers could limit cross-disciplinary information exchange [17,19,21]. Because documentation systems and handover practices differ between hospitals and countries, the specific barriers reported may not apply in the same form elsewhere. Two of the three included studies of structured communication-related activities reported more positive teamwork or safety-culture perceptions [23,25], whereas the third reported mixed findings [35]. These are teamwork-related outcomes, however, and do not establish that communication interventions reduce falls. Future studies should pair process measures with falls and repeat-fall outcomes.

In the included studies, organizational conditions appeared to influence whether interdisciplinary teamwork could be sustained. Participants in 15 studies described workload, staffing, competing priorities, access to training and equipment, leadership, and ward-level champions as constraints on, or enablers of, opportunities for cross-disciplinary interaction [18,19,20,21,22,27,28,29,30,31,32,33,34,36,37]. Although these conditions were described in seven countries, several of the Australian studies were linked to the same falls-prevention programs and research groups, so these conditions may partly reflect particular organizational structures, staffing models, and falls-prevention practices. Research across more diverse hospital systems is needed before judging the transferability of these conditions. Three sources that did not meet this review’s eligibility criteria are noted here for context only and did not contribute to the mapped evidence [40,41,42]. In a study of physical therapists’ perspectives, participants similarly described hospital context, including time constraints, patient volume, staff availability, equipment, and institutional priorities, as constraining falls-prevention practice [40]. A rapid review of nurses’ perspectives reported that nurses identified unit culture and policies, falls education, and communication and collaboration as unit-level barriers and facilitators to implementing recommended falls-prevention strategies [41]. In a community fall-prevention pilot, healthcare providers reported that standardizing interprofessional collaboration required additional effort and that time and financial constraints challenged implementation [42]. Because these sources concern a single professional group or a community setting, their findings should not be assumed to apply directly to interdisciplinary teamwork on acute hospital wards.

The included education and huddle studies also distinguish intervention exposure from teamwork-related outcomes. In one study, collaboration scores did not change significantly after a one-hour education session [26], whereas two studies of structured activities reported more positive teamwork or safety-culture perceptions [23,25]. In a third, team training was followed by improved communication about error, but perceptions of the current state of teamwork decreased, which the authors attributed to greater awareness of gaps in teamwork [35]. These findings do not isolate the effects of duration, frequency, or professional composition, and they do not establish that improving teamwork reduces falls, as none of the four studies that reported falls outcomes reported a statistically significant reduction in falls [23,31,33,35]. Because the methodological quality of these studies was not formally appraised, the reported changes should be read as descriptions of what the included studies found rather than as estimates of intervention effectiveness. Healthcare professionals described patient engagement as requiring coordinated, consistent messages and reported that it could be limited by cognitive impairment, language barriers, and acute illness [17,18,22], while patients in one study also described pain as a barrier [22]. These findings suggest a need for falls-prevention approaches that support consistent communication among professionals, patients, and families while accounting for patients’ clinical and communication needs.

### Implications for Practice, Management, and Education

Although the evidence is descriptive, the mapped studies suggest several implications. For practice, documentation and fall-risk communication that are shared across disciplines may reduce the information gaps described in several studies [17,19,29,37], and structured forums such as post-fall huddles and interdisciplinary handovers may provide regular opportunities for joint discussion of falls prevention [19,23,27,37]. Clearer definition of each discipline’s role in fall-risk assessment and intervention may also help to address the uncertainty about responsibility reported across settings [17,28,29,30,32]. For management, protected time, adequate staffing, visible leadership, and the sharing of unit-level falls data with the whole team appeared to support interdisciplinary engagement [20,22,27,29,30,31,32,33]. For education, the included studies favored repeated, case-based, and interprofessional education over single sessions [18,20,26], and one study noted that team-training sessions did not always include an interdisciplinary group of participants [35], suggesting that the composition of education sessions also warrants attention, which is consistent with the emphasis of the World Health Organization framework on interprofessional education as a route to collaborative practice [8] and with team-training approaches such as TeamSTEPPS [39]. Because the wider evidence on interprofessional collaboration interventions remains limited and heterogeneous [9], these implications should be tested in future evaluations that measure both teamwork processes and falls outcomes.

## 5. Strengths and Limitations

To the authors’ knowledge, this is the first scoping review to systematically map healthcare professionals’ perspectives on interdisciplinary teamwork specifically for falls prevention in hospital settings. The closest published review, a qualitative synthesis of nursing and care staff strategies to prevent older people from falling in health care facilities, identified articulation of the health care team as one complementary strategy but focused on nursing and care staff rather than on interdisciplinary teamwork [43]. The review was conducted using the Arksey and O’Malley framework, as adapted by Levac et al. and modified for JBI methodology, and was reported in accordance with the PRISMA-ScR guidelines. Qualitative, quantitative, and mixed-methods study designs were included to provide a broad overview of the evidence landscape, including perceptual and attitudinal data and quantitative measures of teamwork and safety culture. The inclusion criteria were applied uniformly, and all 21 included studies were conducted in hospital inpatient settings and included participants from two or more professional disciplines or specifically addressed interdisciplinary dynamics. Two reviewers independently screened the titles and abstracts of records from the database searches and reference lists and independently assessed all full texts, with disagreements resolved by discussion and consultation with a third reviewer. The evidence base included studies from eight countries and studies associated with two large falls prevention research programs (the Safe Recovery Programme and the 6-PACK program), offering insight into how interdisciplinary teamwork was perceived across different hospital types and program contexts, although nine of the 21 studies were conducted in Australia.

Several limitations relate to the review process. The inclusion of only English and Arabic language publications may have missed evidence published in other languages and may have introduced selection bias, because studies from health systems in which other languages predominate, and in which interdisciplinary roles and falls-prevention practices may differ, were less likely to be identified. Local implementation reports, service evaluations, and unpublished studies may not have been captured because grey literature was not searched. This is particularly relevant to this review, because implementation reports and service evaluations are where interdisciplinary teamwork in falls prevention is often described, so practice-based accounts of teamwork may be under-represented. Relevant studies indexed only in databases other than the five searched, such as Embase, Scopus, Web of Science, AMED, LILACS, or SciELO, may also have been missed. No Arabic-language database was searched, and forward citation searching was not undertaken, so Arabic-language studies and other studies not indexed in the selected databases may also have been missed. Because terms such as interdisciplinary, interprofessional, and multidisciplinary are used inconsistently in the literature, and three databases were searched with free-text terms only, some relevant studies using other terminology may not have been identified. In addition, the database search strategies were not conceptually identical. The use of an older-adult concept in place of the health-professional concept in PsycINFO may have reduced the sensitivity of that search for studies that did not describe patient age in indexed fields. The date limits applied in MEDLINE and CINAHL began on 1 July 2013 and ended on 31 July 2025. The supplementary MEDLINE search covered 2013 to 2025 in full, but studies published outside these dates and indexed only in CINAHL may not have been retrieved. Studies published in 2026 were outside the eligibility period and were not included, so the most recent evidence may not be represented. Checking the reference lists of included studies identified two eligible studies and one companion report of an included study that the database searches had not retrieved, including one study published in April 2013 [28], and the supplementary MEDLINE search identified nine further eligible studies, which confirms that the original search strategy was not fully sensitive, although the supplementary search was limited to MEDLINE. Combining four concepts, including the health-professional concept, increased the specificity of the search but may have reduced its sensitivity, and terms such as patient safety, safety culture, multifactorial, and interprofessional education were used only in the supplementary MEDLINE search, while the truncated term fall* was used only in PsycINFO and in the supplementary search. Screening also required the involvement of more than one healthcare profession rather than evidence of collaborative processes, so some included studies may reflect multidisciplinary participation rather than interdisciplinary teamwork as defined in this review. No theoretical framework guided the development of the descriptive categories, which were derived inductively and interpreted against the World Health Organization framework and the TeamSTEPPS model only after analysis, and teamwork, communication, and safety culture were therefore not separated analytically. Data were extracted by one author, and although a second reviewer checked all extracted variables against the original articles and the categories were developed by two authors, initial coding by one author may have influenced how findings were categorized. Agreement between reviewers was reached by consensus, but a formal agreement statistic was not calculated. In several included studies, interdisciplinary teamwork was examined within broader evaluations of falls-prevention implementation or education rather than as the primary focus, so the evidence on teamwork from these studies was often partial. A formal critical appraisal was not conducted, in line with the mapping purpose of a scoping review; the findings therefore describe the range and nature of the evidence rather than the methodological certainty of the evidence [12,14]. Because risk of bias and methodological quality were not assessed, and the quantitative findings came from small observational, uncontrolled pre-post, or quasi-experimental designs, these findings should not be interpreted as evidence that any intervention was effective. The eligibility period was extended from 2023 to 2025. The age criterion was refined from adults aged 65 years and over to older adults, a supplementary sensitivity search of MEDLINE was added after the main search, and the review was registered retrospectively on the Open Science Framework. These deviations from the original protocol are reported transparently.

There are also limitations associated with the evidence mapped. Only 21 studies fulfilled the eligibility criteria; the majority were qualitative or mixed-methods studies, and nine were set in Australian hospitals. Several of the Australian studies were also linked to the same falls-prevention programs, notably the Safe Recovery Programme and 6-PACK, and to overlapping research groups, which limits the diversity of the evidence mapped; only one included study was conducted in the Middle East, and none in Africa or in South or East Asia. Results might therefore not be transferable to other healthcare systems with different organizational structures, staffing configurations, job roles, information systems, falls-prevention practices, and cultures. In addition, most included studies did not restrict their patient population by age, and some included younger inpatients, so the findings may partly reflect falls prevention for adult inpatients more generally. The relatively small evidence base is itself an important finding, because empirical studies directly exploring how healthcare professionals experience and implement interdisciplinary teamwork for hospital falls prevention are limited.

## 6. Conclusions

This scoping review set out to map the existing literature on healthcare professionals’ perspectives on interdisciplinary teamwork for falls prevention among older adults in hospital settings. The relatively small evidence base, predominantly qualitative and mixed-methods and with nine of the 21 studies conducted in Australian hospitals, allows only tentative answers to the review question. Healthcare professionals from multiple disciplines generally regard interdisciplinary teamwork as important for falls prevention, but this commitment is not consistently translated into routine, shared practice. Across the four descriptive categories identified by this review, interdisciplinary teamwork was shaped less by the stated value of collaboration than by the conditions that enabled it. Responsibility frequently remained centered on nursing, while role ambiguity limited mutual understanding of professional contributions. Separate documentation systems, variable handovers and transfers between wards, and inconsistent messaging could prevent information from becoming coordinated action. Workload and staffing pressures, limited access to training and equipment, and inadequate leadership support further appeared to influence whether collaboration could be sustained.

Within the settings studied, the mapped literature indicates that interdisciplinary teamwork is more likely to be sustained when role clarification, shared information systems, opportunities for cross-disciplinary dialogue, leadership support, and patient engagement are built into routine ward practice. Although these conditions were described across eight countries, nine of the 21 included studies were conducted in Australian hospitals, so they may not transfer directly to health systems with different organizational structures, professional roles, staffing models, documentation systems, and falls-prevention practices. Structured activities, including post-fall huddles and interdisciplinary education, were associated with more positive perceptions of teamwork and safety culture in two studies [23,25], whereas team training was followed by lower perceptions of the current state of teamwork, which the authors attributed to greater awareness [35]. None of the four studies that reported falls outcomes reported a statistically significant reduction in falls [23,31,33,35], so the available studies did not establish that these activities themselves reduce falls, nor did they isolate the effects of duration, frequency, or professional composition. Patient participation is inseparable from this work, as consistent messages from multiple professional groups can support engagement, but cognitive impairment, pain, language barriers, and acute illness may limit patients’ capacity to participate. Future research should assess how specific interdisciplinary teamwork arrangements are implemented and sustained in diverse hospital systems. Mixed-methods studies combining measures of role clarity, communication, teamwork, and safety culture with falls and repeat-fall outcomes, alongside comparative studies across countries and hospital types, would help clarify which organizational conditions convert a shared aspiration for falls prevention into reliable interdisciplinary practice.

## Figures and Tables

**Table 1:** Search strategy for MEDLINE via EBSCOhost.

ID	Search Strategy	Results
#1	(MH “Interprofessional Relations+”) OR (MH “Patient Care Team+”) OR TI (interprofessional OR interdisciplinary OR multidisciplinary OR “interprofessional collaboration” OR “interdisciplinary collaboration” OR “multidisciplinary collaboration” OR “interprofessional teamwork” OR “interdisciplinary teamwork” OR “multidisciplinary teamwork” OR “interprofessional team*” OR “interdisciplinary team*” OR “multidisciplinary team*” OR “collaborative practice”) OR AB (interprofessional OR interdisciplinary OR multidisciplinary OR “interprofessional collaboration” OR “interdisciplinary collaboration” OR “multidisciplinary collaboration” OR “interprofessional teamwork” OR “interdisciplinary teamwork” OR “multidisciplinary teamwork” OR “interprofessional team*” OR “interdisciplinary team*” OR “multidisciplinary team*” OR “collaborative practice”)	176,780
#2	(MH “Accidental Falls+”) OR TI (“fall prevention” OR “falls prevention” OR “fall risk” OR “falls risk” OR “fall prevention program*” OR “fall prevention intervention*” OR “fall risk assessment” OR “falls risk assessment”) OR AB (“fall prevention” OR “falls prevention” OR “fall risk” OR “falls risk” OR “fall prevention program*” OR “fall prevention intervention*” OR “fall risk assessment” OR “falls risk assessment”)	19,557
#3	(MH “Hospitals+”) OR (MH “Inpatients+”) OR TI (hospital* OR inpatient* OR “acute care” OR “rehabilitation ward*” OR “geriatric unit*”) OR AB (hospital* OR inpatient* OR “acute care” OR “rehabilitation ward*” OR “geriatric unit*”)	1,136,167
#4	(MH “Health Personnel+”) OR TI (“healthcare professional*” OR “health professional*” OR nurse* OR physician* OR doctor* OR physiotherapist* OR “physical therapist*” OR “occupational therapist*” OR pharmacist* OR “social worker*”) OR AB (“healthcare professional*” OR “health professional*” OR nurse* OR physician* OR doctor* OR physiotherapist* OR “physical therapist*” OR “occupational therapist*” OR pharmacist* OR “social worker*”)	689,162
#5	#1 AND #2 AND #3 AND #4	107

Note. MH = Medical Subject Heading, where + indicates that narrower headings were included; TI = title; AB = abstract; * = truncation.

## Data Availability

All data supporting the findings of this review are contained within the article and its Supplementary Materials, including the search strategies (Table S1), the summary of quantitative evidence (Table S2), the data-charting form (Table S3), and the completed PRISMA-ScR checklist (Table S4 ).

## Supplementary Materials

The following supporting information can be downloaded at: https://www.mdpi.com/article/10.3390/healthcare14213658/s1. Table S1: Search strategies for all databases. Table S2: Summary of quantitative evidence from the included studies. Table S3: Data-charting form. Table S4: Preferred Reporting Items for Systematic reviews and Meta-Analyses extension for Scoping Reviews (PRISMA-ScR) Checklist.

## Abbreviations

The following abbreviations are used in this manuscript:

AHRQ HSOPSC	Agency for Healthcare Research and Quality Hospital Survey on Patient Safety Culture;
CIHC	Canadian Interprofessional Health Collaborative;
COM-B	capability, opportunity, motivation, and behavior model;
DONs	Directors of Nursing;
i-PARIHS	integrated Promoting Action on Research Implementation in Health Services framework;
IPE	interprofessional education;
JBI	Joanna Briggs Institute;
MDT	multidisciplinary team;
NHS	National Health Service;
NUMs	Nurse Unit Managers;
OSF	Open Science Framework;
OT	occupational therapy;
PCC	Population, Concept and Context;
PRISMA-ScR	Preferred Reporting Items for Systematic Reviews and Meta-Analyses extension for Scoping Reviews;
PT	physiotherapy;
QI	quality improvement;
RN	registered nurse;
RPN	registered practical nurse;
SRP	Safe Recovery Programme;
STEADI	Stopping Elderly Accidents, Deaths, and Injuries;
TeamSTEPPS	Team Strategies and Tools to Enhance Performance and Patient Safety.
