# Supplementary material for: Healthcare Professionals’ Perspectives on Interdisciplinary Teamwork for Falls Prevention Among Older Adults in Hospital Settings: A Scoping Review
Source: Healthcare (Basel). 2026 Oct 28;14(21):3658. doi: 10.3390/healthcare14213658 (PMC13762671; doi:10.3390/healthcare14213658)
Supplement: Supplementary file 1 [file healthcare-14-03658-s001.zip › healthcare-4581736-supplementary.pdf]

Table S1. Search strategies for all databases, searched on 10 August 2026, and for the supplementary MEDLINE sensitivity search, conducted on 2 October 2026

MEDLINE via EBSCOhost

**ID**	MEDLINE via EBSCOhost	**Results**
**#1**	(MH "Interprofessional Relations+") OR (MH "Patient Care Team+") OR TI (interprofessional OR interdisciplinary OR multidisciplinary OR "interprofessional collaboration" OR "interdisciplinary collaboration" OR "multidisciplinary collaboration" OR "interprofessional teamwork" OR "interdisciplinary teamwork" OR "multidisciplinary teamwork" OR "interprofessional team*" OR "interdisciplinary team*" OR "multidisciplinary team*" OR "collaborative practice") OR AB (interprofessional OR interdisciplinary OR multidisciplinary OR "interprofessional collaboration" OR "interdisciplinary collaboration" OR "multidisciplinary collaboration" OR "interprofessional teamwork" OR "interdisciplinary teamwork" OR "multidisciplinary teamwork" OR "interprofessional team*" OR "interdisciplinary team*" OR "multidisciplinary team*" OR "collaborative practice")	176,780
**#2**	(MH "Accidental Falls+") OR TI ("fall prevention" OR "falls prevention" OR "fall risk" OR "falls risk" OR "fall prevention program*" OR "fall prevention intervention*" OR "fall risk assessment" OR "falls risk assessment") OR AB ("fall prevention" OR "falls prevention" OR "fall risk" OR "falls risk" OR "fall prevention program*" OR "fall prevention intervention*" OR "fall risk assessment" OR "falls risk assessment")	19,557
**#3**	(MH "Hospitals+") OR (MH "Inpatients+") OR TI (hospital* OR inpatient* OR "acute care" OR "rehabilitation ward*" OR "geriatric unit*") OR AB (hospital* OR inpatient* OR "acute care" OR "rehabilitation ward*" OR "geriatric unit*")	1,136,167
**#4**	(MH "Health Personnel+") OR TI ("healthcare professional*" OR "health professional*" OR nurse* OR physician* OR doctor* OR physiotherapist* OR "physical therapist*" OR "occupational therapist*" OR pharmacist*	689,162

	OR "social worker*") OR AB ("healthcare professional*" OR "health professional*" OR nurse* OR physician* OR doctor* OR physiotherapist* OR "physical therapist*" OR "occupational therapist*" OR pharmacist* OR "social worker*")	
**#5**	**#1 AND #2 AND #3 AND #4**	**107**

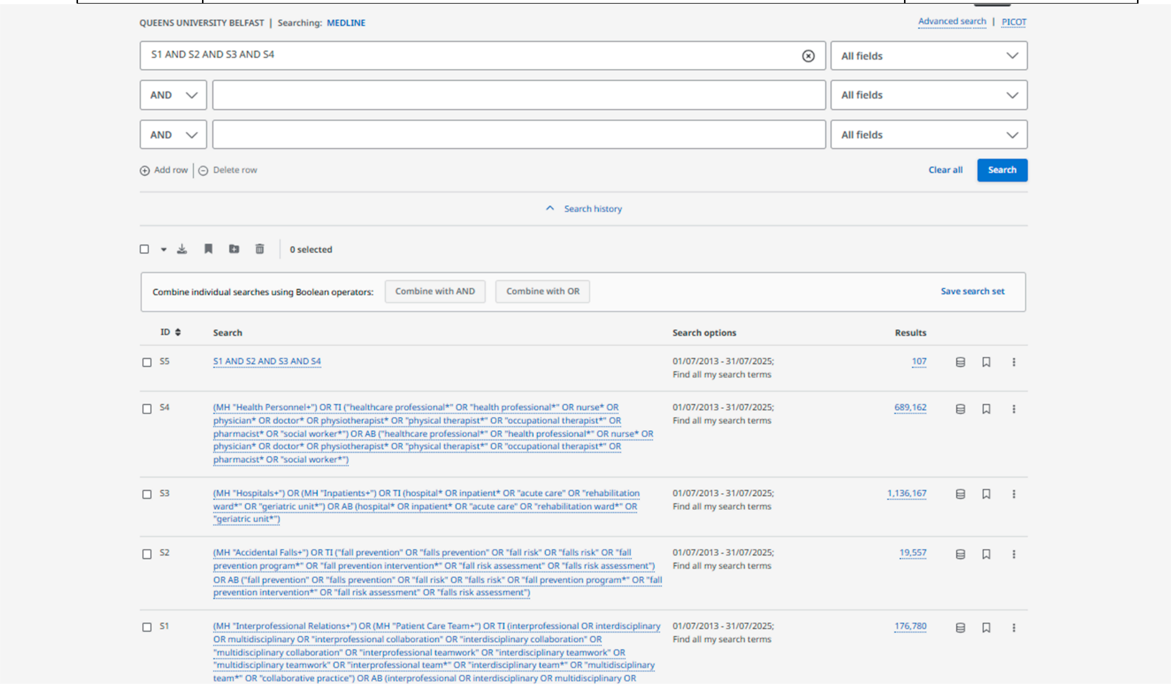

CINAHL via EBSCOhost

Platform EBSCOhost, CINAHL database. Searched on 10 August 2026. Every line was limited to publication dates 01/07/2013 to 31/07/2025, with the search mode Find all my search terms and the option Apply equivalent subjects. MH = CINAHL subject heading, + = exploded, TI = title, AB = abstract. No language or publication-type limits were applied.

**ID**	CINAHL via EBSCOhost	**Results**
#1	(MH "Interdisciplinary Communication" OR MH "Patient Care Team" OR MH "Health Care Teams" OR TI (interprofessional OR interdisciplinary OR multidisciplinary OR "interprofessional collaboration" OR "interdisciplinary collaboration" OR "multidisciplinary collaboration" OR "interprofessional teamwork" OR "interdisciplinary teamwork" OR "multidisciplinary teamwork" OR "interprofessional team*" OR "interdisciplinary team*" OR "multidisciplinary team*" OR	60,253

	"collaborative practice") OR AB (interprofessional OR interdisciplinary OR multidisciplinary OR "interprofessional collaboration" OR "interdisciplinary collaboration" OR "multidisciplinary collaboration" OR "interprofessional teamwork" OR "interdisciplinary teamwork" OR "multidisciplinary teamwork" OR "interprofessional team*" OR "interdisciplinary team*" OR "multidisciplinary team*" OR "collaborative practice"))	
#2	(MH "Accidental Falls" OR TI ("fall prevention" OR "falls prevention" OR "fall risk" OR "falls risk" OR "fall prevention program*" OR "fall prevention intervention*" OR "fall risk assessment" OR "falls risk assessment") OR AB ("fall prevention" OR "falls prevention" OR "fall risk" OR "falls risk" OR "fall prevention program*" OR "fall prevention intervention*" OR "fall risk assessment" OR "falls risk assessment"))	17,862
#3	(MH "Hospitals+" OR MH "Inpatients" OR TI (hospital* OR inpatient* OR "acute care" OR "rehabilitation ward*" OR "geriatric unit*") OR AB (hospital* OR inpatient* OR "acute care" OR "rehabilitation ward*" OR "geriatric unit*"))	438,358
#4	(MH "Health Personnel+" OR TI ("healthcare professional*" OR "health professional*" OR nurse* OR physician* OR doctor* OR physiotherapist* OR "physical therapist*" OR "occupational therapist*" OR pharmacist* OR "social worker*") OR AB ("healthcare professional*" OR "health professional*" OR nurse* OR physician* OR doctor* OR physiotherapist* OR "physical therapist*" OR "occupational therapist*" OR pharmacist* OR "social worker*"))	565,856
**#5**	**#1 AND #2 AND #3 AND #4**	**95**

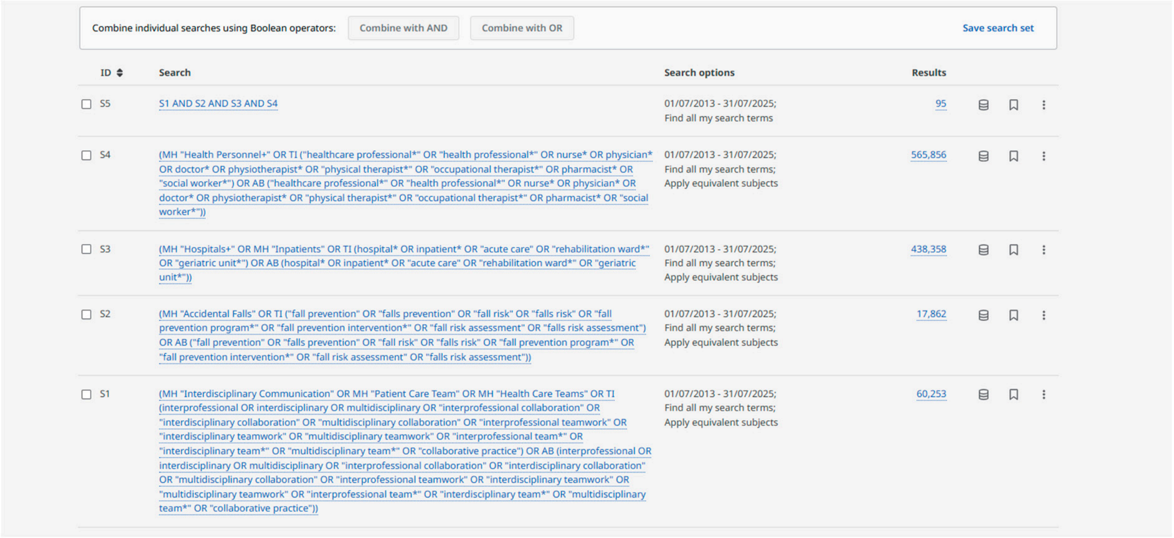

Combine individual searches using Boolean operators: Combine with AND Combine with OR Save search set

ID	Search	Search options	Results
S5	S1 AND S2 AND S3 AND S4	01/07/2013 - 31/07/2025; Find all my search terms	95
S4	(MH "Health Personnel+" OR TI ("healthcare professional*" OR "health professional*" OR nurse* OR physician* OR doctor* OR physiotherapist* OR "physical therapist*" OR "occupational therapist*" OR pharmacist* OR "social worker*") OR AB ("healthcare professional*" OR "health professional*" OR nurse* OR physician* OR doctor* OR physiotherapist* OR "physical therapist*" OR "occupational therapist*" OR pharmacist* OR "social worker*"))	01/07/2013 - 31/07/2025; Find all my search terms; Apply equivalent subjects	565,856
S3	(MH "Hospitals+" OR MH "Inpatients" OR TI (hospital* OR inpatient* OR "acute care" OR "rehabilitation ward*" OR "geriatric unit*") OR AB (hospital* OR inpatient* OR "acute care" OR "rehabilitation ward*" OR "geriatric unit*"))	01/07/2013 - 31/07/2025; Find all my search terms; Apply equivalent subjects	438,358
S2	(MH "Accidental Falls" OR TI ("fall prevention" OR "falls prevention" OR "fall risk" OR "falls risk" OR "fall prevention program*" OR "fall prevention intervention*" OR "fall risk assessment" OR "falls risk assessment") OR AB ("fall prevention" OR "falls prevention" OR "fall risk" OR "falls risk" OR "fall prevention program*" OR "fall prevention intervention*" OR "fall risk assessment" OR "falls risk assessment"))	01/07/2013 - 31/07/2025; Find all my search terms; Apply equivalent subjects	17,862
S1	(MH "Interdisciplinary Communication" OR MH "Patient Care Team" OR MH "Health Care Teams" OR TI (interprofessional OR interdisciplinary OR multidisciplinary OR "interprofessional collaboration" OR "interdisciplinary collaboration" OR "multidisciplinary collaboration" OR "interprofessional teamwork" OR "interdisciplinary teamwork" OR "multidisciplinary teamwork" OR "interprofessional team*" OR "interdisciplinary team*" OR "multidisciplinary team*" OR "collaborative practice") OR AB (interprofessional OR interdisciplinary OR multidisciplinary OR "interprofessional collaboration" OR "interdisciplinary collaboration" OR "multidisciplinary collaboration" OR "interprofessional teamwork" OR "interdisciplinary teamwork" OR "multidisciplinary teamwork" OR "interprofessional team*" OR "interdisciplinary team*" OR "multidisciplinary team*" OR "collaborative practice"))	01/07/2013 - 31/07/2025; Find all my search terms; Apply equivalent subjects	60,253

PsycINFO via Ovid

Platform Ovid, APA PsycInfo database. Searched on 10 August 2026. Free-text terms were searched in the Ovid multi-purpose field (.mp = title, abstract, heading word, table of contents, key concepts, original title, tests and measures, mesh word), and no APA Thesaurus subject headings were exploded. Lines 2, 5, and 10 repeat lines 1, 4, and 9, and lines 3, 6, 8, and 11 limit lines 2, 5, 7, and 10 to publication years 2013 to 2025. Line 12 combines line 2 with the year-limited lines 6, 8, and 11, so all retrieved records fall within 2013 to 2025. No language or publication-type limits were applied.

**ID**	PsycINFO via Ovid	**Results**
#1	(interprofession* or interdisciplin* or multidisciplin* or multiprofession* or teamwork or "team work" or "team-based" or "team based" or collaborat* or "interprofessional practice" or "interdisciplinary practice" or "multidisciplinary practice" or "interprofessional collaboration" or "interdisciplinary collaboration" or "multidisciplinary collaboration" or "interprofessional team*" or "interdisciplinary team*" or "multidisciplinary team*" or "healthcare team*" or "health care team*" or "patient care team*" or "shared responsibility" or "shared decision*").mp. [mp=title, abstract, heading word, table of contents, key concepts, original title, tests & measures, mesh word]	214,274
#2	(interprofession* or interdisciplin* or multidisciplin* or multiprofession* or teamwork or "team work" or "team-based" or "team based" or collaborat* or "interprofessional practice" or "interdisciplinary	214,274

	practice" or "multidisciplinary practice" or "interprofessional collaboration" or "interdisciplinary collaboration" or "multidisciplinary collaboration" or "interprofessional team*" or "interdisciplinary team*" or "multidisciplinary team*" or "healthcare team*" or "health care team*" or "patient care team*" or "shared responsibility" or "shared decision*").mp. [mp=title, abstract, heading word, table of contents, key concepts, original title, tests & measures, mesh word]	
#3	limit 2 to yr="2013 - 2025"	119,461
#4	(fall* or "accidental fall*" or "fall prevention" or "falls prevention" or "fall risk" or "falls risk" or "fall prevention program*" or "fall prevention intervention*" or "fall risk assessment" or "falls risk assessment" or "fall-related injur*" or "fall related injur*").mp.	66,643
#5	(fall* or "accidental fall*" or "fall prevention" or "falls prevention" or "fall risk" or "falls risk" or "fall prevention program*" or "fall prevention intervention*" or "fall risk assessment" or "falls risk assessment" or "fall-related injur*" or "fall related injur*").mp.	66,643
#6	limit 5 to yr="2013 - 2025"	31,712
#7	(hospital* or inpatient* or "acute care" or "acute hospital*" or "hospital setting*" or "hospital ward*" or "medical ward*" or "surgical ward*" or "rehabilitation ward*" or "geriatric ward*" or "geriatric unit*" or "inpatient setting*").mp.	283,360
#8	limit 7 to yr="2013 - 2025"	128,259
#9	(older adult* or older people or older person* or elderly or elder* or aged or senior* or geriatric* or gerontolog* or "later life" or "later life adult*").mp.	832,548
#10	(older adult* or older people or older person* or elderly or elder* or aged or senior* or geriatric* or gerontolog* or "later life" or "later life adult*").mp.	832,548
#11	limit 10 to yr="2013 - 2025"	328,559

**#12**	**2 and 6 and 8 and 11**	**100**

Search History (13) View Saved

#	Searches	Results	Type	Runtime	Actions	Annotations
1	(interprofession* or interdisciplin* or multidisciplin* or multiprofession* or teamwork or "team work" or "team-based" or "team based" or collaborat* or "interprofessional practice" or "interdisciplinary practice" or "multidisciplinary practice" or "interprofessional collaboration" or "interdisciplinary collaboration" or "multidisciplinary collaboration" or "interprofessional team*" or "interdisciplinary team*" or "multidisciplinary team*" or "healthcare team*" or "health care team*" or "patient care team*" or "shared responsibility" or "shared decision*").mp. [mp=title, abstract, heading word, table of contents, key concepts, original title, tests & measures, mesh word]	214274	Advanced	0.00	Display Results More	
2	(interprofession* or interdisciplin* or multidisciplin* or multiprofession* or teamwork or "team work" or "team-based" or "team based" or collaborat* or "interprofessional practice" or "interdisciplinary practice" or "multidisciplinary practice" or "interprofessional collaboration" or "interdisciplinary collaboration" or "multidisciplinary collaboration" or "interprofessional team*" or "interdisciplinary team*" or "multidisciplinary team*" or "healthcare team*" or "health care team*" or "patient care team*" or "shared responsibility" or "shared decision*").mp. [mp=title, abstract, heading word, table of contents, key concepts, original title, tests & measures, mesh word]	214274	Advanced	0.00	Display Results More	
3	limit 2 to yr="2013 - 2025"	119461	Advanced	0.22	Display Results More	
4	(fall* or "accidental fall*" or "fall prevention" or "falls prevention" or "fall risk" or "falls risk" or "fall prevention program*" or "fall prevention intervention*" or "fall risk assessment" or "falls risk assessment" or "fall-related injur*" or "fall related injur*").mp.	66643	Advanced	4.81	Display Results More	
5	(fall* or "accidental fall*" or "fall prevention" or "falls prevention" or "fall risk" or "falls risk" or "fall prevention program*" or "fall prevention intervention*" or "fall risk assessment" or "falls risk assessment" or "fall-related injur*" or "fall related injur*").mp.	66643	Advanced	0.00	Display Results More	
6	limit 5 to yr="2013 - 2025"	31712	Advanced	0.13	Display Results More	
7	(hospital* or inpatient* or "acute care" or "acute hospital*" or "hospital setting*" or "hospital ward*" or "medical ward*" or "surgical ward*" or "rehabilitation ward*" or "geriatric ward*" or "geriatric unit*" or "inpatient setting*").mp.	283360	Advanced	4.23	Display Results More	
8	limit 7 to yr="2013 - 2025"	128259	Advanced	0.20	Display Results More	
9	(older adult* or older people or older person* or elderly or elder* or aged or senior* or geriatric* or gerontolog* or "later life" or "later life adult*").mp.	832548	Advanced	3.55	Display Results More	
10	(older adult* or older people or older person* or elderly or elder* or aged or senior* or geriatric* or gerontolog* or "later life" or "later life adult*").mp.	832548	Advanced	0.00	Display Results More	
11	limit 10 to yr="2013 - 2025"	328559	Advanced	0.29	Display Results More	
12	2 and 6 and 8 and 11	100	Advanced	0.03	Display Results More	

Cochrane Library

Platform Cochrane Library (Wiley). Searched on 10 August 2026. Each line was searched in the title, abstract, and keyword fields (ti,ab,kw) with word variations, and no publication date limit was applied (Cochrane Library publication date to present). Publication date was therefore assessed during screening. No language or publication-type limits were applied.

**ID**	**Cochrane Library**	Results
**#1**	(interprofessional OR interdisciplinary OR multidisciplinary OR "interprofessional collaboration" OR "interdisciplinary collaboration" OR "multidisciplinary collaboration" OR "interprofessional teamwork" OR "interdisciplinary teamwork" OR "multidisciplinary teamwork" OR interprofessional team* OR interdisciplinary team* OR multidisciplinary team* OR "collaborative practice"):ti,ab,kw	14,943
**#2**	("fall prevention" OR "falls prevention" OR "fall risk" OR "falls risk" OR fall prevention program* OR fall prevention intervention* OR "fall risk assessment" OR "falls risk assessment" OR "Accidental Falls"):ti,ab,kw	7,888
**#3**	(hospital* OR inpatient* OR "acute care" OR rehabilitation ward* OR geriatric unit*):ti,ab,kw	305,487

#4	(healthcare professional* OR health professional* OR nurse* OR physician* OR doctor* OR physiotherapist* OR physical therapist* OR occupational therapist* OR pharmacist* OR social worker*):ti,ab,kw	152,485
**#5**	**#1 AND #2 AND #3 AND #4**	**67**

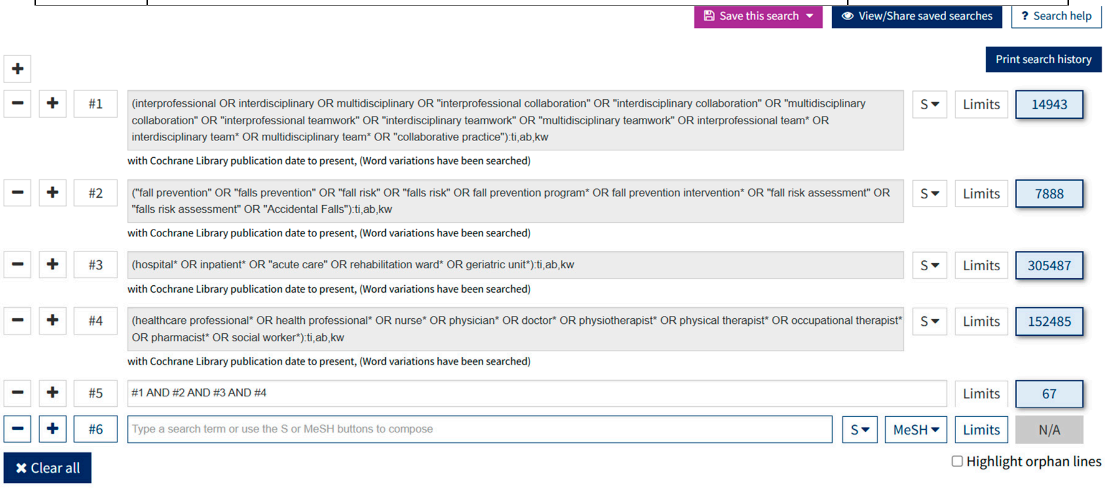

Europe PMC

Platform Europe PMC advanced search. Searched on 10 August 2026. Lines 1 and 2 were restricted to title and abstract (TITLE_ABS), whereas lines 3 and 4 were run without a field restriction and therefore searched all fields. No publication date limit was applied, and publication date was therefore assessed during screening. No language or publication-type limits were applied.

ID	Europe PMC	Results
**#1**	TITLE_ABS:(interprofessional OR interdisciplinary OR multidisciplinary OR "interprofessional collaboration" OR "interdisciplinary collaboration" OR "multidisciplinary collaboration" OR "interprofessional teamwork" OR "interdisciplinary teamwork" OR "multidisciplinary teamwork" OR "interprofessional team" OR "interdisciplinary team" OR "multidisciplinary team" OR "collaborative practice")	272,128
**#2**	TITLE_ABS:("fall prevention" OR "falls prevention" OR "fall risk" OR "falls risk" OR "fall prevention program" OR "fall prevention programs" OR "fall prevention intervention" OR "fall prevention interventions" OR "fall risk assessment" OR "falls risk assessment" OR "Accidental Falls")	15,221

**#3**	(hospital* OR inpatient* OR "acute care" OR "rehabilitation ward" OR "rehabilitation wards" OR "geriatric unit" OR "geriatric units")	11,138,905
**#4**	("healthcare professional" OR "healthcare professionals" OR "health professional" OR "health professionals" OR nurse* OR physician* OR doctor* OR physiotherapist* OR "physical therapist" OR "physical therapists" OR "occupational therapist" OR "occupational therapists" OR pharmacist* OR "social worker" OR "social workers")	4,265,914
**#5**	**#1 AND #2 AND #3 AND #4**	**295**

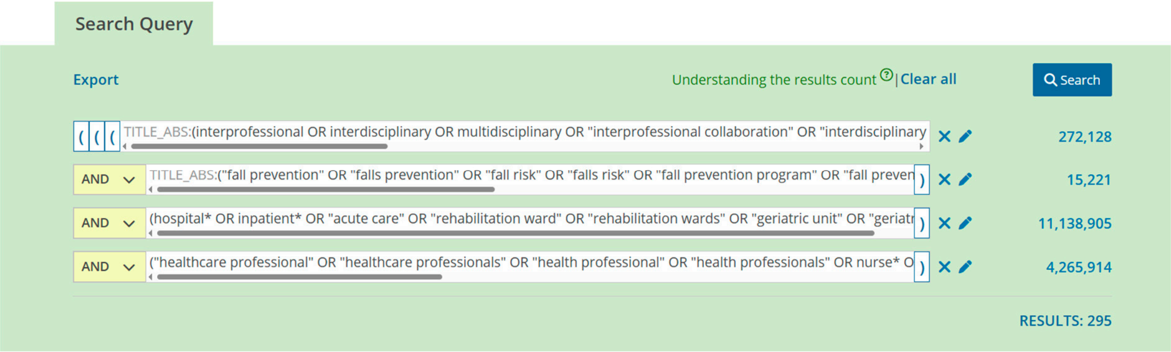

Supplementary sensitivity search: MEDLINE via Ovid

Platform Ovid, Ovid MEDLINE(R) ALL database (1946 to October 01, 2026). Searched on 2 October 2026. Compared with the main MEDLINE search, the health-professional concept was omitted, the truncated term fall* was used, and terms for patient safety, safety culture, multifactorial interventions, and interprofessional education were added. exp = exploded subject heading, / = subject heading, .ti,ab. = title or abstract, or/ = combination of lines with OR. Line 14 limits line 13 to publication years 2013 to 2025. No language or publication-type limits were applied.

**ID**	MEDLINE via Ovid	**Results**
#1	exp Accidental Falls/	32,332
#2	fall*.ti,ab.	293,576
#3	1 or 2	300,407
#4	exp Patient Care Team/	77,829
#5	exp Interprofessional Relations/	75,122

#6	Patient Safety/	29,377
#7	(interdisciplinar* or interprofessional or inter-professional or multidisciplinar* or multi-disciplinar* or teamwork or "team work" or collaborat* or "patient safety" or "safety culture" or multifactorial or "interprofessional education").ti,ab.	643,540
#8	or/4-7	753,943
#9	exp Hospitals/	344,722
#10	Inpatients/	34,397
#11	(hospital* or inpatient* or ward* or "acute care").ti,ab.	2,117,697
#12	or/9-11	2,230,633
#13	3 and 8 and 12	3,318
**#14**	**limit 13 to yr="2013 - 2025"**	**2,221**

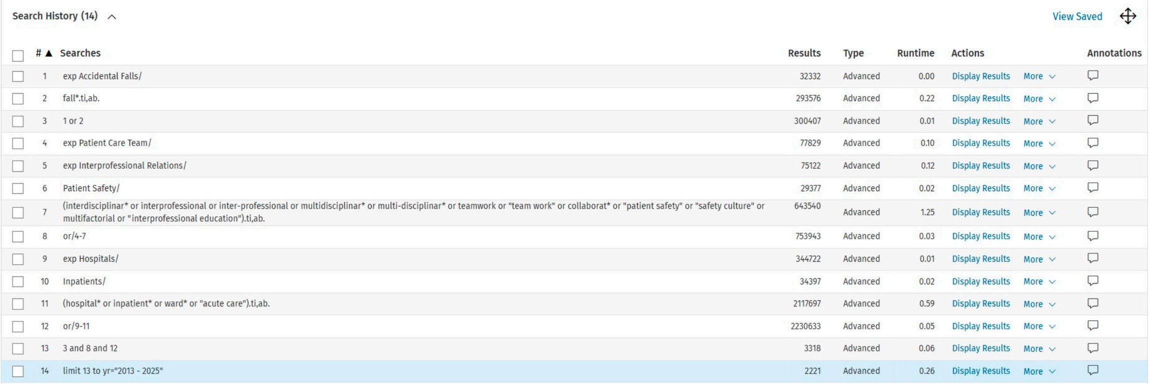

Search History (14)

#	Searches	Results	Type	Runtime	Actions	Annotations
1	exp Accidental Falls/	32332	Advanced	0.00	Display Results More	
2	fall*.ti,ab.	293576	Advanced	0.22	Display Results More	
3	1 or 2	300407	Advanced	0.01	Display Results More	
4	exp Patient Care Team/	77829	Advanced	0.10	Display Results More	
5	exp Interprofessional Relations/	75122	Advanced	0.12	Display Results More	
6	Patient Safety/	29377	Advanced	0.02	Display Results More	
7	(interdisciplinar* or interprofessional or inter-professional or multidisciplinar* or multi-disciplinar* or teamwork or "team work" or collaborat* or "patient safety" or "safety culture" or multifactorial or "interprofessional education").ti,ab.	643540	Advanced	1.25	Display Results More	
8	or/4-7	753943	Advanced	0.03	Display Results More	
9	exp Hospitals/	344722	Advanced	0.01	Display Results More	
10	Inpatients/	34397	Advanced	0.02	Display Results More	
11	(hospital* or inpatient* or ward* or "acute care").ti,ab.	2117697	Advanced	0.59	Display Results More	
12	or/9-11	2230633	Advanced	0.05	Display Results More	
13	3 and 8 and 12	3318	Advanced	0.06	Display Results More	
14	limit 13 to yr="2013 - 2025"	2221	Advanced	0.26	Display Results More	

View Saved

Search totals

Database	Platform	Date searched	Publication date limit	Records retrieved
MEDLINE	EBSCOhost	10 August 2026	01/07/2013 to 31/07/2025	107
CINAHL	EBSCOhost	10 August 2026	01/07/2013 to 31/07/2025	95
APA PsycInfo	Ovid	10 August 2026	2013 to 2025	100

Cochrane Library	Wiley	10 August 2026	None, assessed at screening	67
Europe PMC	Europe PMC	10 August 2026	None, assessed at screening	295
**Total before deduplication**				**664**
Supplementary sensitivity search: MEDLINE	Ovid	2 October 2026	2013 to 2025	2,221

The total of 664 records corresponds to the records identified in the PRISMA-ScR flow diagram (Figure 1) of the manuscript. The supplementary MEDLINE sensitivity search was conducted separately from the main search, and its 2,221 records are not included in the total of 664. Its results are reported in Section 3.1 and in the PRISMA-ScR flow diagram (Figure 1) of the manuscript. Language and publication type were not limited in any search. After deduplication, 45 records were removed because of an ineligible publication type or a language other than English or Arabic.

Table S2

Summary of quantitative evidence from the included studies

Study	Study design	Sample size	Professional groups	Intervention/exposure	Measurement instrument	Outcome	Main result	Major methodological limitation
Ayton et al. (2017) [20]	Multicenter mixed-methods pre-implementation study, with a nurse survey, focus groups, and key informant interviews structured by the COM-B framework; survey data analyzed descriptively	Survey 420 of 702 returned (60%); 12 focus groups with 96 nurses; 24 senior staff interviews (540 participants in total)	Survey respondents mainly registered nurses (74%); focus groups with nurses; interviews with 6 Directors of Nursing, 7 Nurse Unit Managers, 8 nursing educators, a clinical risk coordinator, a quality and safety manager, and a clinical program nurse manager	None. Perceptions were collected before implementation of the nurse-led 6-PACK program	42-item nurse survey rated on a 5-point Likert scale, with 7 items mapped to the COM-B domains of capability, opportunity, and motivation	Beliefs about falls prevention and perceived barriers and enablers to implementation	80% of nurses believed they were responsible for falls prevention; 46% disagreed that older people cannot be stopped from falling (23% undecided); 32% felt they received useful training from falls prevention leaders; 42% agreed there was strong ward leadership support for falls prevention (35% neutral). No statistical testing	Descriptive survey data only; perceptions collected before implementation; perspectives of doctors and allied health professionals not captured; participating wards volunteered for the trial, which the authors noted may have introduced selection bias
Jones et al. (2019) [23]	Quasi-experimental evaluation, with a longitudinal hospital-level analysis of repeat falls and cross-sectional staff surveys at study end	16 hospitals; 347 falls in 308 patients. Teamwork survey 814 of 1649 invited (49.4%); safety-culture survey 1843 of 2761 (66.8%)	Direct-care staff, members of interprofessional coordinating teams (nursing, pharmacy, physical and/or occupational therapy, quality improvement), providers, and administrators	Post-fall huddles introduced with training, a pocket guide, and a documentation form over two years. Exposure was the proportion of falls followed by a huddle (hospital level) and participation in at least one huddle (staff level)	Repeat fall rate (falls divided by unique patients who fell); adapted TeamSTEPPS Teamwork Perceptions Questionnaire (35 items); AHRQ Hospital Survey on Patient Safety Culture (42 items)	Repeat fall rate; percent positive teamwork and safety-culture perceptions	Inverse correlation between the proportion of falls followed by a huddle and repeat fall rate ($r = -.47$, $p = .07$, not significant). Huddle participants reported more positive perceptions than non-participants, for example teamwork across hospital departments 75% versus 66% positive ($p = .011$) and team leadership 91% versus 82% ($p < .001$). No confidence intervals reported	Hospital-level correlation based on 16 hospitals; staff-level comparison of self-selected huddle participants and non-participants at a single time point; self-reported measures; possible ceiling effect noted by the authors

Study	Study design	Sample size	Professional groups	Intervention/exposure	Measurement instrument	Outcome	Main result	Major methodological limitation
Lopez-Jeng and Eberth (2020) [25]	Uncontrolled pre–post evaluation, with surveys 1 month before and 30 days after the education, plus post-session quizzes	Safety-culture survey 78 pre-test and 40 post-test respondents (separate anonymous convenience samples); 12 to 14 attendees per lesson	Administration, dietetics, patient care assistants, pharmacy, resident physicians, registered and licensed nurses, technicians, therapists, and unit clerical staff	Four weekly 1-hour interdisciplinary education lessons based on the Five As behavior change model, delivered by the hospital falls-prevention committee	Modified AHRQ Hospital Survey on Patient Safety Culture; 13-item true or false knowledge quiz and self-rated knowledge, attitudes, and motivation after each lesson	Percent positive safety-culture composite scores; post-session knowledge and self-ratings	Improvement in 10 of 11 safety-culture composites, significant for management support (+12.0 points, p = .014), overall perceptions of patient safety (+12.8, p = .005), teamwork across units (+11.6, p = .010), and handoffs and transitions (+13.0, p = .008). No confidence intervals reported and statistical test not named	No control group; different pre-test and post-test samples with a changed staff mix; a new falls-reporting system was introduced during the project; knowledge measured after sessions only
Shaw et al. (2023) [26]	Mixed methods, with pre–post surveys compared by independent samples t-tests and semi-structured telephone interviews	34 pre-test and 25 post-test survey respondents; 7 interviewees	Registered nurses (21), enrolled nurses (9), physiotherapists (2), occupational therapist (1), unknown (1); interviews also included an assistant nurse unit manager and an executive	A single 1-hour interprofessional education session, face to face or via Zoom, on implementing a modified Safe Recovery Programme	Interprofessional Collaborative Competency Attainment Survey (20 items); 8-item survey on evidence-based falls education; 7-item evaluation of the session	Self-rated interprofessional collaboration competency; views on falls education	No statistically significant change on any of the 20 collaboration items (all p ≥ .12). One falls-education item improved, feeling well prepared to educate patients (mean 3.76 to 4.20, p = .007). Means reported with 95% confidence intervals	Single short session; unpaired pre-test and post-test groups; predominantly nursing sample; high baseline ratings; no falls outcomes measured
Milisen et al. (2013) [28]	Descriptive multicenter feasibility study, with a questionnaire completed after a one-month test of a multidisciplinary fall prevention guideline	49 healthcare workers from 17 geriatric wards in 17 hospitals; 512 patients screened	Geriatricians (7), nurses (13), physiotherapists (12), occupational therapists (17)	A multidisciplinary fall prevention guideline covering case finding, multifactorial assessment, targeted interventions, and transfer of information at discharge, introduced through a 2-hour training session	Questionnaire with 27 structured and 7 open questions for individual healthcare workers, based on five levels of implementation barriers and facilitators	Perceived usefulness, feasibility, capability, responsibility, and barriers to implementation	For most risk assessments and interventions, several disciplines considered themselves responsible and capable. Barriers included time investment (81.1%), lack of communication between disciplines (35.8%), insufficient multidisciplinary teamwork (28.3%), and lack of interest from hospital management (15.4%). Descriptive statistics only for these items	Descriptive post-test questionnaire only; one-month test period; small numbers per discipline; no statistical testing of these perceptions

Study	Study design	Sample size	Professional groups	Intervention/exposure	Measurement instrument	Outcome	Main result	Major methodological limitation
Spiva et al. (2014) [35]	Quasi-experimental, longitudinal repeated-measures design with intervention and comparison groups	71 consented; 34 completed all three time points (18 intervention, 16 comparison); 54 unit-level observations	Recruitment pool of registered nurses, pharmacists, physical therapists, and physicians; registered nurses were the largest group of completers (29.4%)	Falls-prevention team training based on TeamSTEPPS, with video vignettes, debriefing, and reinforcement	AHRQ Hospital Survey on Patient Safety Culture; TeamSTEPPS Teamwork Attitudes Questionnaire; TeamSTEPPS Teamwork Perceptions Questionnaire; structured observations; fall and injury rates	Safety culture, teamwork attitudes and perceptions, observed interprofessional communication, falls, and fall injuries	In the intervention group, feedback and communication about error improved over time ($F = 4.95$, $p < .01$), and teamwork perceptions decreased ($F = 3.92$, $p = .03$), which the authors attributed to greater awareness of the gap between best practice and day-to-day teamwork. Teamwork attitudes improved in the whole sample ($F = 3.240$, $p = .04$). Differences between groups were not significant. Falls fell from 2.69 to 1.03 per 1,000 patient-days ($p = .15$), not statistically significant	Small final sample with attrition; training sessions did not always include an interdisciplinary group; concurrent roll-out of a new fall risk assessment tool
Stuby et al. (2025) [36]	Mixed-methods process evaluation of a quality improvement intervention	E-learning completed by 94 of 139 residents and 229 of 405 nurses; survey completed by 13% of residents and 18% of nurses; 25 interviews	Residents and registered nurses (interviews: 17 nurses, 8 residents)	E-learning, oral presentation, and reminder quizzes on fall risk assessment and prevention	Online survey with Likert-scale and free-text items; semi-structured interviews	Participation, acceptability, and perceived impact on practice and on interprofessional collaboration	About one quarter of clinicians reported an impact of the intervention on interprofessional collaboration (descriptive). E-learning completion differed between the two hospitals (86% versus 45%, $p < .001$)	Low survey participation and possible reporting bias; findings on interprofessional collaboration were descriptive only
Toye et al. (2019) [37]	Mixed methods, including staff surveys, nurse focus groups, environmental audits, and administrative data	105 staff survey respondents; 21 focus-group participants	Survey: nurses (69%), medical staff (8.6%), allied health professionals (8.6%), and others; focus groups: nurses	None. Interprofessional collaboration was assessed on four medical wards	Assessment of Interprofessional Team Collaboration Scale	Partnership, cooperation, coordination, and total collaboration scores	Mean scores across wards were 77.6 of 95 for partnership, 45.3 of 55 for cooperation, 27.1 of 35 for coordination, and 150.1 of 185 in total, interpreted as good collaboration (descriptive)	Descriptive survey data only; relatively low survey response; single hospital

Note. Only the eight included studies that reported quantitative findings relevant to interdisciplinary teamwork are shown. Quantitative findings reported by Macchiavello et al. [31], McLennan et al. [32], and Ploeg et al. [33] concerned participant feedback, acceptability, awareness, or fall rates rather than interdisciplinary teamwork and were not charted.

Heng et al. [27], a companion report of Shaw et al. [26], also reported patient survey data that did not relate to interdisciplinary teamwork and were not charted. Randell et al. [21] also reported a descriptive review of 60 patient records on the documentation of falls risk assessments and care plans, and routinely collected falls counts presented as contextual information only. These data did not relate to interdisciplinary teamwork and were not charted. Percentage-point differences for Lopez-Jeng and Eberth are differences between pre-test and post-test percent positive scores. No quantitative synthesis was undertaken, and these findings should not be interpreted as estimates of intervention effectiveness. AHRQ = Agency for Healthcare Research and Quality; COM-B = capability, opportunity, motivation, behavior.

Table S3

Data-charting form

Field	Variable	Content charted
1	Author(s)	Authors as listed in the source
2	Year of publication	Year of the journal issue
3	Country	Country in which the study was conducted
4	Study aim	Aim or research question as stated by the authors
5	Study design	Design and methods of data collection
6	Setting	Hospital type and number of hospitals, wards, or units
7	Sample size	Number of participants, by method of data collection where reported
8	Participant disciplines	Professional groups included. Patient or family participants were recorded separately
9	Analytical approach	Qualitative, quantitative, or mixed-methods analysis, including any framework or statistical tests used
10	Key findings relevant to interdisciplinary teamwork in falls prevention	Findings relating to interdisciplinary teamwork in falls prevention, with illustrative quotations, percentages, effect estimates, confidence intervals, and p-values where reported

Note. The form was based on the JBI data extraction tool and piloted on two included studies by two reviewers (AA and GM). Data were charted for all included studies, one author (AA) checked all charted data against the original articles, and a second reviewer (GM) independently checked all charted variables against the original articles. The patient population as reported, the evidence type, and the descriptive categories shown in Table 2 were recorded during analysis. Companion reports of the same study were charted together.

Table S4 Preferred Reporting Items for Systematic reviews and Meta-Analyses extension for Scoping Reviews (PRISMA-ScR) Checklist

SECTION	ITEM	PRISMA-ScR CHECKLIST ITEM	REPORTED ON PAGE #
**TITLE**			
Title	1	Identify the report as a scoping review.	Page 1
**ABSTRACT**			
Structured summary	2	Provide a structured summary that includes (as applicable): background, objectives, eligibility criteria, sources of evidence, charting methods, results, and conclusions that relate to the review questions and objectives.	Page 1
**INTRODUCTION**			

SECTION	ITEM	PRISMA-ScR CHECKLIST ITEM	REPORTED ON PAGE #
Rationale	3	Describe the rationale for the review in the context of what is already known. Explain why the review questions/objectives lend themselves to a scoping review approach.	Pages 2–3
Objectives	4	Provide an explicit statement of the questions and objectives being addressed with reference to their key elements (e.g., population or participants, concepts, and context) or other relevant key elements used to conceptualize the review questions and/or objectives.	Pages 3–4
**METHODS**			
Protocol and registration	5	Indicate whether a review protocol exists; state if and where it can be accessed (e.g., a Web address); and if available, provide registration information, including the registration number.	Pages 3–4
Eligibility criteria	6	Specify characteristics of the sources of evidence used as eligibility criteria (e.g., years considered, language, and publication status), and provide a rationale.	Pages 4–5
Information sources*	7	Describe all information sources in the search (e.g., databases with dates of coverage and contact with authors to identify additional sources), as well as the date the most recent search was executed.	Page 5
Search	8	Present the full electronic search strategy for at least 1 database, including any limits used, such that it could be repeated.	Pages 5–6; Supplementary Table S1
Selection of sources of evidence†	9	State the process for selecting sources of evidence (i.e., screening and eligibility) included in the scoping review.	Page 6
Data charting process‡	10	Describe the methods of charting data from the included sources of evidence (e.g., calibrated forms or forms that have been tested by the team before their use, and whether data charting was done independently or in duplicate) and any processes for obtaining and confirming data from investigators.	Pages 6–7

SECTION	ITEM	PRISMA-ScR CHECKLIST ITEM	REPORTED ON PAGE #
Data items	11	List and define all variables for which data were sought and any assumptions and simplifications made.	Pages 6–7
Critical appraisal of individual sources of evidence§	12	If done, provide a rationale for conducting a critical appraisal of included sources of evidence; describe the methods used and how this information was used in any data synthesis (if appropriate).	N/A
Synthesis of results	13	Describe the methods of handling and summarizing the data that were charted.	Page 7
**RESULTS**			
Selection of sources of evidence	14	Give numbers of sources of evidence screened, assessed for eligibility, and included in the review, with reasons for exclusions at each stage, ideally using a flow diagram.	Pages 8–9 (Figure 1)
Characteristics of sources of evidence	15	For each source of evidence, present characteristics for which data were charted and provide the citations.	Pages 10–17 (Table 2)
Critical appraisal within sources of evidence	16	If done, present data on critical appraisal of included sources of evidence (see item 12).	N/A
Results of individual sources of evidence	17	For each included source of evidence, present the relevant data that were charted that relate to the review questions and objectives.	Pages 11–23 (Table 2 and Section 3.3); Supplementary Table S2
Synthesis of results	18	Summarize and/or present the charting results as they relate to the review questions and objectives.	Pages 18–23
**DISCUSSION**			
Summary of evidence	19	Summarize the main results (including an overview of concepts, themes, and types of evidence available), link to the review questions and objectives, and consider the relevance to key groups.	Pages 24–26

SECTION	ITEM	PRISMA-ScR CHECKLIST ITEM	REPORTED ON PAGE #
Limitations	20	Discuss the limitations of the scoping review process.	Pages 26–28
Conclusions	21	Provide a general interpretation of the results with respect to the review questions and objectives, as well as potential implications and/or next steps.	Pages 28–29
**FUNDING**			
Funding	22	Describe sources of funding for the included sources of evidence, as well as sources of funding for the scoping review. Describe the role of the funders of the scoping review.	Page 29

JBI = Joanna Briggs Institute; PRISMA-ScR = Preferred Reporting Items for Systematic reviews and Meta-Analyses extension for Scoping Reviews.
* Where *sources of evidence* (see second footnote) are compiled from, such as bibliographic databases, social media platforms, and Web sites.
† A more inclusive/heterogeneous term used to account for the different types of evidence or data sources (e.g., quantitative and/or qualitative research, expert opinion, and policy documents) that may be eligible in a scoping review as opposed to only studies. This is not to be confused with *information sources* (see first footnote).
‡ The frameworks by Arksey and O'Malley (6) and Levac and colleagues (7) and the JBI guidance (4, 5) refer to the process of data extraction in a scoping review as data charting.
§ The process of systematically examining research evidence to assess its validity, results, and relevance before using it to inform a decision. This term is used for items 12 and 19 instead of "risk of bias" (which is more applicable to systematic reviews of interventions) to include and acknowledge the various sources of evidence that may be used in a scoping review (e.g., quantitative and/or qualitative research, expert opinion, and policy document).

From: Tricco AC, Lillie E, Zarin W, O'Brien KK, Colquhoun H, Levac D, et al. PRISMA Extension for Scoping Reviews (PRISMAScR): Checklist and Explanation. Ann Intern Med. 2018;169:467–473. doi: 10.7326/M18-0850.
